# Testing the molecular clock using mechanistic models of fossil preservation and molecular evolution

**DOI:** 10.1098/rspb.2017.0227

**Published:** 2017-06-21

**Authors:** Rachel C. M. Warnock, Ziheng Yang, Philip C. J. Donoghue

**Affiliations:** 1 School of Earth Sciences, University of Bristol, Life Sciences Building, Tyndall Avenue, Bristol BS8 1TQ, UK; 2 Department of Paleobiology, National Museum of Natural History, The Smithsonian Institution, Washington, DC 20560, USA; 3 Department of Biosystems Science and Engineering, ETH Zürich, Mattenstrasse 26, 4058 Basel, Switzerland; 4 Department of Genetics, Evolution and Environment, University College London, Darwin Building, Gower Street, London WC1E 6BT, UK

**Keywords:** fossil record, sampling bias, Bayesian phylogenetics, molecular clock, MCMCTREE

## Abstract

Molecular sequence data provide information about relative times only, and fossil-based age constraints are the ultimate source of information about absolute times in molecular clock dating analyses. Thus, fossil calibrations are critical to molecular clock dating, but competing methods are difficult to evaluate empirically because the true evolutionary time scale is never known. Here, we combine mechanistic models of fossil preservation and sequence evolution in simulations to evaluate different approaches to constructing fossil calibrations and their impact on Bayesian molecular clock dating, and the relative impact of fossil versus molecular sampling. We show that divergence time estimation is impacted by the model of fossil preservation, sampling intensity and tree shape. The addition of sequence data may improve molecular clock estimates, but accuracy and precision is dominated by the quality of the fossil calibrations. Posterior means and medians are poor representatives of true divergence times; posterior intervals provide a much more accurate estimate of divergence times, though they may be wide and often do not have high coverage probability. Our results highlight the importance of increased fossil sampling and improved statistical approaches to generating calibrations, which should incorporate the non-uniform nature of ecological and temporal fossil species distributions.

## 1. Introduction

The fossil record formerly provided the only time scale for evolutionary history, despite the combined phylogenetic, ecological and stratigraphic processes that have resulted in a highly incomplete and non-uniform record of life [1]. Molecular clock dating has superseded the role of the fossil record in establishing the age for many clades [2]. However, molecular sequences are only informative about the genetic distance between species (the expected number of substitutions); that is, the *relative* age of clades—estimating absolute ages requires a clock model and temporal calibration information. Hence, calibration of the molecular clock relies ultimately on information derived from fossil evidence (or other geological events). Fossil data therefore remain integral to most molecular clock analyses.

Uncertainty in Bayesian divergence time estimates can be broadly attributed to (i) having finite amounts of sequence data and (ii) uncertainty in the calibrations [3–5], even if the correct sequence-evolution model has been specified. Empirical studies have often found that much of the uncertainty in divergence time estimates is due to uncertainty in the calibrations [3,6]. Indeed, different ways of representing fossil data as the prior probability of clade ages can lead to dramatic differences in divergence estimates [7–12]. This has led to controversy about how, or even if, palaeontological data should be used to date the Tree of Life [13–16], in addition to attempts to reduce uncertainty using whole-genome data [17–21]. Despite the well-recognized importance of fossil calibrations in molecular clock dating, it has not been possible to assess the accuracy of fossil calibration methods or molecular divergence estimates based on empirical data alone, as the true divergence times are unknown. However, these questions can be approached through simulation.

Previous simulation-based attempts to assay the performance of molecular clock methods have not accommodated the variables that affect the stratigraphic distribution of fossils. Here, we conduct simulations that combine mechanistic models of fossil preservation and molecular sequence evolution, and demonstrate the utility of this framework in testing the accuracy and precision of Bayesian species divergence time estimation. A major challenge to constructing reliable clade age constraints is that the stratigraphic distribution of fossils is highly uneven, influenced by factors that lead to variation in sedimentary rock volume during different intervals. We incorporate such variation into our simulations using a model that relates the probability of fossil recovery (the combined effects of preservation and sampling) to cyclic changes in sea level [22]. Simulated fossil data were then used to construct calibrations using the three main heuristic approaches, allowing us to assess the relative importance of increased sampling of fossils versus genetic loci. We show that increased sampling of both fossil and molecular data increases the accuracy and precision of posterior divergence times, but accuracy and precision are ultimately driven by the calibrations. We demonstrate that the performance of competing approaches will be determined by the distribution of fossils relative to divergence times, which is influenced by tree shape, preservation model and, in particular, fossil recovery rates. Finally, the result of a molecular clock analysis is commonly reported using the mean or median of the posterior time estimate, along with the 95% Bayesian credible intervals. We demonstrate that at realistic levels of fossil sampling, the mean or median will be a poor approximation of the true result, because the uncertainty associated with divergence time estimates will be great. The posterior credible interval is a more accurate, if not precise, age estimate. The results of our simulation study suggest that controls on the stratigraphic distribution of fossil taxa, and their sampling, should inform the development of models for divergence time analysis.

## 2. Material and methods

### (a) Simulation of fossil occurrence and sequence data

Stratigraphic occurrences of fossils were simulated for two trees of 16 extant taxa, one balanced and one unbalanced, under uniform and non-uniform models of preservation. The use of fixed topologies makes the interpretation of results more straightforward than random trees generated from the birth–death process. The time period between the age of the root (100 Ma) and the present was divided into 50 equal stratigraphic intervals. One hundred million years are treated as one time unit. During each interval, *p* is the probability of sampling any given lineage*.* Here, *p* reflects the joint effects of preservation potential and sampling intensity, which are indistinguishable in such a model. Under the uniform model, *p* is simply equal to the specified sampling intensity *s*. To simulate non-uniform occurrence data, we used a model of preservation [22,23] that uses water depth as a proxy for preservation or sampling potential in the marine stratigraphic record. Sampling probability is given by2.1
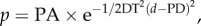
where *d* is the current water depth, PD the preferred depth, DT the depth tolerance and PA the peak abundance. Water depth was simulated using the sine wave function2.2
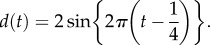


**Figure 1.**
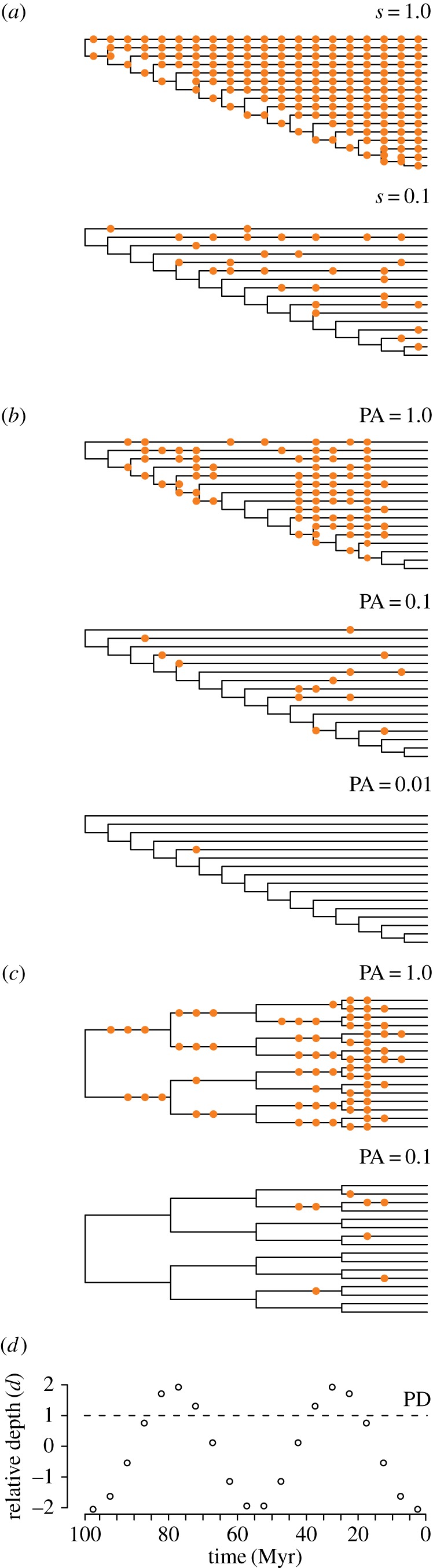
Example simulated fossil data under uniform and non-uniform models of preservation on balanced and unbalanced trees. In (*a*), the tree is fully unbalanced and preservation is uniform. The probability of sampling during each interval is equal to the sampling intensity (*s*)*.* In (*b*), the tree is fully unbalanced, and preservation is non-uniform. The probability of sampling during each interval is determined as a function of water depth (shown in (*d*)), preferred depth (PD), depth tolerance (DT) and peak abundance (PA). In (*c*), the tree is fully balanced and preservation is non-uniform. (Online version in colour.)

This emulates two successive transgression/regression events over the interval 0–100 Myr, with a range in relative depth of −2 to 2. We used four values of *s* and PA (0.001, 0.01, 0.1 and 1), with PD = 1 and DT = 1 fixed, to reflect the perceived completeness of the fossil record [24,25]. Example datasets of sampled fossils are shown in figure 1.

Each tree was used to generate 100 sequence alignments using the program evolver (PAML 4.8) [26]. We generated data with *L* = 1, 2, 10 or 20 loci, with 1000 bp at each locus. For each locus *i*, an overall mean rate *μ_i_* was sampled from a gamma distribution, *G*(2, 2), with the mean = 1 substitution per site per unit time (10^–8^ substitutions per site per year). Given the overall rate for locus *i*, independent rates for branches on the tree were sampled from a lognormal distribution with the mean rate *μ_i_* and standard deviation of the log rate *σ* = 0.1. This independent rates model allows variable rates both among multiple loci and among branches at each locus. Branch lengths, in expected number of substitutions per site, were calculated as the product of time duration of the branch and rate. The HKY + Γ_5_ substitution model was used to simulate sequences, with equal base frequencies, transition/transversion ratio *κ* = 5 and gamma shape parameter *α* = 0.25 for rate heterogeneity across sites.

### (b) Minimum and maximum constraints on divergence times

The simulated fossil occurrence data were used to establish minimum and maximum constraints on node ages, which were used to construct calibration densities in the Bayesian estimation of divergence times (electronic supplementary material, figure S1). Minimum constraints were based on first appearances and three approaches were used to establish maximum constraints. First, we used a stratigraphic bracketing approach to estimate 95% confidence intervals on stratigraphic ranges [27]. Second, phylogenetic bracketing was used to emulate best-practice approaches of establishing calibrations (e.g. [15,16]). Third, we generated arbitrary maximum bounds to be 110, 125, 150 and 175% of the age of the minimum constraints.

### (c) Calibration densities

We implemented two calibration strategies in the molecular clock dating analyses using MCMCTREE. First, we used the minimum and maximum fossil constraints obtained using stratigraphic and phylogenetic bracketing to generate soft-uniform bounds [5]. We used sharp minimum (tail probability *p*_L_ = = 0.1%) and soft maximum bounds (*p*_U_ = 2.5%). Second, we used the skew-*t* distribution and specified the parameters by attempting to match the minimum and maximum bounds to the 0.1 and 97.5% percentiles of the distribution. The arbitrary maximum bounds were also implemented using the skew-*t* distribution following this approach. We applied a soft-uniform calibration at the root of the tree (*p*_U_ = 2.5%). When there were insufficient data to inform the maximum constraint at the root, this was set to twice the true age for the root (200 Ma). If no fossils were sampled at all, the root age was assigned a uniform distribution over the interval *U*(0, 2).

### (d) Molecular clock analysis

MCMCTREE [26] was used to date species divergences with the sequence alignments using the approximate likelihood method [28]. The proportion of calibrated nodes on the tree varied from 0 to 1: in some datasets, no fossils were sampled and no fossil calibrations were generated, while in other cases, every node had a calibration. A uniform prior on times for the non-calibration nodes was generated from the birth–death sampling process, with parameters *λ* = 1, *μ* = 1 and *ρ* = 0. Maximum-likelihood estimates of branch lengths were calculated using baseml under the HKY + Γ_5_ substitution model.

In the analysis of multi-loci sequence data, we used the gamma-Dirichlet prior [29] on the rates for loci (*μ_i_*), implemented in MCMCTREE. A gamma prior is assigned on the average rate among loci, 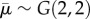 (mean = 1 or 10^–8^ substitutions/site/year), and a uniform Dirichlet distribution is used to partition the total rate for each locus (*μ_i_*,). Given the rate *μ_i_* for locus *i*, the branch rates at the locus are assigned independent lognormal distributions with the variance parameter 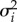. This is the independent rates model. Similarly, the variance parameters (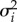) are assigned a gamma-Dirichlet prior, with the average of 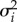 having a gamma prior *G*(1, 10) (mean = 0.1).

Further details of the simulations, MCMC analysis and performance measures are presented in the electronic supplementary material. The experimental design is outlined in electronic supplementary material, figure S2. In total, we performed 64 000 molecular clock analyses. Code used to perform the analysis is available on Dryad: http://dx.doi.org/10.5061/dryad.5706p [30].

## 3. Results

### (a) Under realistic models of fossil preservation, overall calibrations improve with improved sampling

Our main objectives are to examine the accuracy and precision of the fossil calibrations generated using different approaches, and the subsequent posterior time estimates when the calibrations are used in a molecular dating analysis. We considered a fossil calibration to be accurate if the true age fell within the minimum and maximum bounds. The different approaches for constructing calibrations were compared using coverage—the probability that the calibration bounds cover the true age, averaged over nodes and simulated replicates. By this definition of accuracy, calibrations that are so wide as to be effectively uninformative may be accurate nevertheless. We measure the precision of a calibration by the relative interval width [3], also averaged across nodes and replicates.

Minimum fossil-based constraints were based on sampled first appearances, and so the minimum bounds were always younger than the true divergence times. Under the uniform model of preservation, the minima become increasingly closer in age to the true age as the probability of sampling increases. By contrast, under the non-uniform model, the minima do not necessarily improve as sampling increases (figure 1). The accuracy and precision of calibrations inferred using three alternative approaches to deriving maxima—stratigraphic bracketing, phylogenetic bracketing or arbitrary constraints—improved consistently with increased fossil sampling, with the exception of stratigraphic bracketing, which became less accurate with increased sampling when preservation was non-uniform (electronic supplementary material, tables S1–S4). The accuracy and precision of all approaches to deriving calibrations were dependent on (i) preservation model, (ii) sampling intensity and (iii) tree shape. Ultimately, these variables affect the distribution of fossils relative to the true ages. As our goal is to assess the impact of fossil preservation on molecular divergence estimates, we examine in detail the impact of these variables in the subsequent sections and, in particular, focus on the accuracy and precision of the Bayesian priors and posteriors.

### (b) Point estimates are often inaccurate because credible intervals are large

**Figure 2.**
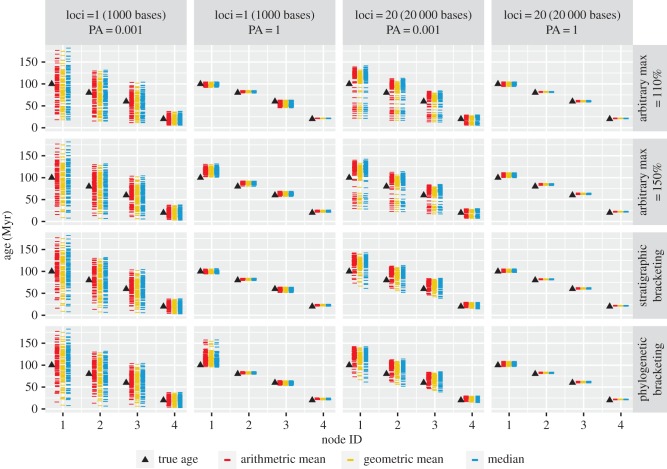
Posterior means (red or yellow), medians (blue) and true node ages (black triangles) are shown for four selected nodes (see electronic supplementary material, figure S3). Results are shown for 100 replicates using the unbalanced tree under the non-uniform model of fossil preservation, given low versus high sampling intensities (PA = 0.001 or 1.0). Calibration methods include arbitrary maxima (at 110 and 150%), stratigraphic bracketing and phylogenetic bracketing, using skew-*t* calibration densities.

Molecular divergence times are typically reported using standard posterior summaries—the mean or median of the posterior distribution, along with the 95% highest posterior density or credible intervals (95% HPDs). Our results suggest that the posterior means and medians of node ages are often poor estimates of true ages, partly because the intervals are wide (figure 2; electronic supplementary material, figure S4). By contrast, the 95% HPDs are more likely to contain the true divergence time. This is particularly important in cases where sequence sampling and especially fossil sampling is low or the calibrations are imprecise. However, when the amount of data is large and the results converge on the wrong answer, the posteriors may be precise but fail to encompass the correct clade age (i.e. they are inaccurate). In these cases, both the mean and the 95% HPD intervals will provide a poor approximation of clade age. Therefore, any comparison between competing methods should consider both accuracy and precision.

We explored the impact of competing variables on prior and posterior estimates of divergence times using coverage (the proportion of HPDs that contain the true age), relative interval width (the width of the HPD intervals) and relative root mean square error (RMSE), which is a combined measure of accuracy and precision. When coverage is used to define accuracy, a very wide interval, though uninformative, is accurate because it encompasses the true age. We place emphasis on the RMSE as a combined measure of accuracy and precision, but first illustrate how coverage can be misleading.

### (c) Coverage can be worse in the posterior than the prior when the prior intervals are very wide

**Figure 3.**
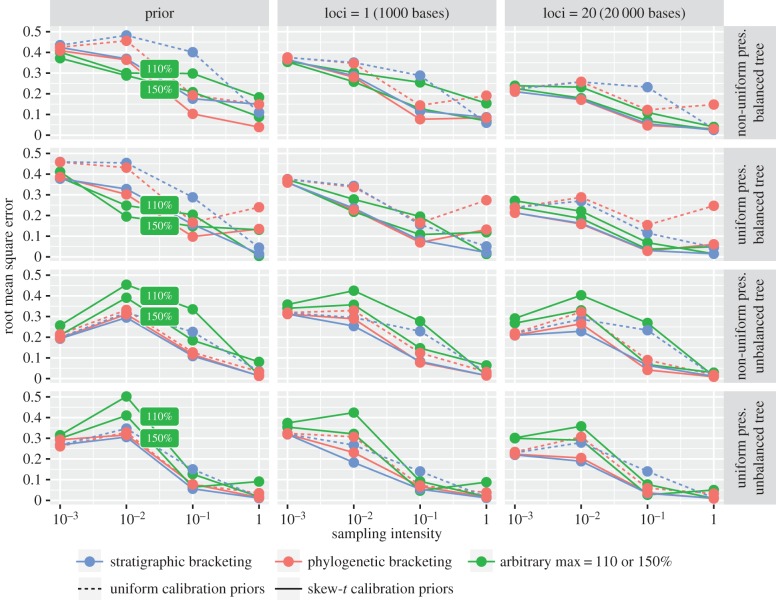
Average RMSE for the ages in datasets simulated under different conditions. Sampling intensity is PA and *s* under the non-uniform and uniform models of fossil preservation, respectively. Coloured lines show the results obtained for different calibration approaches: arbitrary maxima (at 110 and 150%), stratigraphic bracketing and phylogenetic bracketing. Each point represents the normalized RMSE averaged over nodes and replicates.

**Figure 4.**
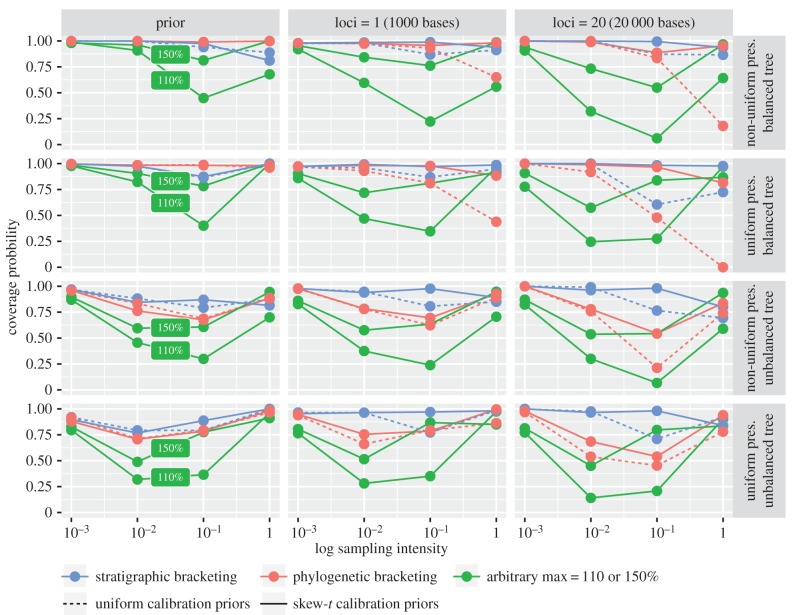
Average coverage probability of the 95% HPD intervals of divergence times for datasets simulated under different conditions. Sampling intensity is PA and *s* under the non-uniform and uniform models of fossil preservation, respectively. Coloured lines show the results obtained for different calibration approaches: arbitrary maxima (at 110 and 150%), stratigraphic bracketing and phylogenetic bracketing. Coverage probability is averaged over nodes and replicates.

The overall patterns obtained for the coverage, precision and RMSE values for the priors were reflected in the posteriors, demonstrating the strength of the relationship between the priors and posteriors (figures 3 and 4; electronic supplementary material, figure S5). The choice of uniform versus skew-*t* calibration densities also had a large impact on the performance of stratigraphic and phylogenetic bracketing, with the skew-*t* producing higher coverage and lower RMSE values, and in some cases shorter intervals (figures 3 and 4; electronic supplementary material, figure S5 and tables S1–S4). Stratigraphic bracketing produced constraints with good coverage (= 0.88–1.0) under both models of preservation, but resulted in a larger range of coverage in both the prior (uniform densities: 0.79–1.0; skew-*t* densities: 0.77–1.0) and posterior (uniform: 0.6–1.0; skew-*t*: 0.8–1.0). Phylogenetic bracketing produced constraints with reasonable coverage (=0.6–1.0) and a similar range in the prior (uniform: 0.69–1.0, skew-*t*: 0.68–1.0), but produced a much larger range in the posterior (uniform: 0.0–1.0; skew-*t*: 0.54–1.0). Thus, coverage in the posterior can be worse than in the prior. This occurs when the prior intervals are very wide, relative to the posterior intervals, and the true node age lies close to the bounds of the 95% prior density. This highlights the importance of considering interval width together with coverage.

The RMSE demonstrates that the skew-*t* calibration density consistently produced more accurate and precise results than did uniform calibration densities, and in some cases, the difference was considerable (figure 3). However, fossil sampling had an even greater impact, and increased sampling improved results across all methods, irrespective of the calibration density (with the exception of phylogenetic bracketing, fully balanced tree).

### (d) Preservation scenario and fossil sampling drive the accuracy and precision of prior and posterior divergence time estimates

Alternative preservation scenarios had a large impact on prior and posterior divergence estimates (figures 3 and 4; electronic supplementary material, figure S5 and tables S1–S4). Although overall results were similar under both models of preservation (the median RMSE was 0.19 for non-uniform and 0.22 for uniform preservation), the results were impacted strongly by sampling intensity. The results obtained under the uniform model of preservation were more precise than those obtained under non-uniform preservation (median HPD width: 0.38 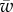 versus 0.53 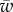; electronic supplementary material, figure S5). This appears to be because the sampled fossils tend to cover the whole temporal range under the uniform model of preservation, while under the non-uniform model, some intervals often did not contain any fossils (figure 1).

Increased fossil sampling led to a consistent decrease in interval width for the priors and posteriors under both models of preservation (electronic supplementary material, figure S5), and led to an overall increase in accuracy, in terms of both RMSE and coverage, with some exceptions (figures 3 and 4). In some cases, as sampling increases, the results get worse before improving with the addition of more fossil data. This is because, at the lowest sampling level (*s*, PA = 0.001), fossils are rarely sampled and so results are dominated by the diffuse calibration density on the root age (0 < *t* < 2). The posterior intervals were wide but cover the true age. Although the results are more accurate at high rates of fossil sampling (*s*, PA = 1) relative to intermediate rates (*s*, PA = 0.01 or 0.1), this was not consistent across methods and depended on other variables, such as tree topology. Even given the best-case sampling scenario (*s*, PA = 1), the coverage of most methods was less than 95% (electronic supplementary material, tables S1–S4).

### (e) Accuracy and precision of molecular clock estimates vary with tree shape

Tree shape had a large impact on the relative performance of competing approaches to calibration (figures 3 and 4; electronic supplementary material, tables S1–S4). For equivalent preservation scenarios, the balanced and unbalanced trees resulted in different estimates of RMSE and coverage for competing calibration approaches (figures 3 and 4). Tree shape also had an impact on the overall interval width (the median prior interval width was 1.29 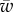 for the balanced versus 0.72 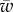 for the unbalanced tree; median posterior width: 0.51 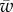 versus 0.38 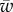), which may also be attributable to the greater degree of overlap between the constraints in the unbalanced tree. These results may be attributable to two factors: (i) the unbalanced tree contains a larger number of nested (or hierarchical) nodes, so that truncation has a greater impact than in the balanced tree, and (ii) the unbalanced tree contains longer internal branches, which increases the potential for large gaps between divergence times and first appearances, especially given non-uniform preservation (figure 1). However, the overall results are similar for the balanced and unbalanced trees (the median posterior RMSE was 0.18 for the balanced versus 0.21 for the unbalanced tree), including the positive impact of fossil sampling.

### (f) Adding sequence data increases accuracy and precision, but accuracy and precision is ultimately determined by the calibrations

The addition of 20-fold sequence data led to an overall improvement in accuracy and precision, as reflected by the RMSE estimates (figure 3). Across competing calibration methods, the average difference in RMSE between the priors and posteriors was −6% based on the analyses of one locus (1000 bases), and −34% based on the analyses of 20 loci (20 000 bases; in the case of RMSE, a negative change is desirable). The average difference in RMSE between the posteriors obtained using one versus 20 loci was −31%. However, the average difference in RMSE between the posteriors obtained using 10 versus 20 loci was only −6%.

**Figure 5.**
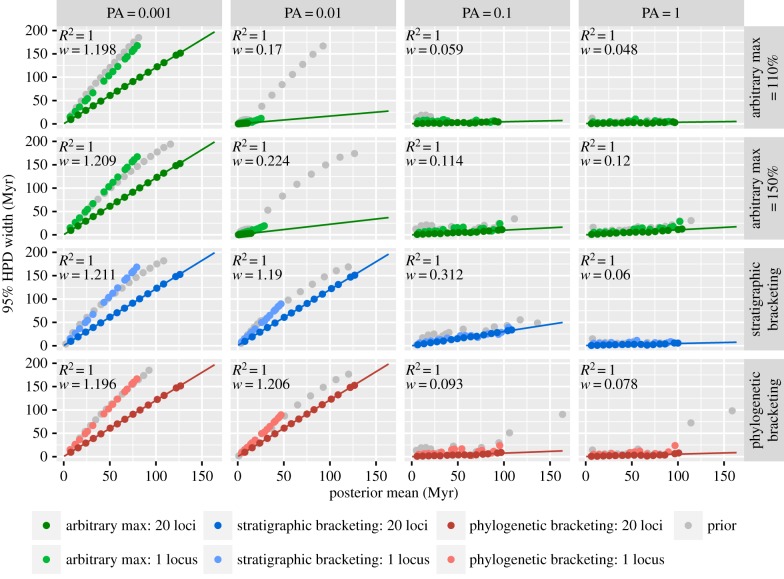
Infinite-sites plots for data simulated on the unbalanced tree under the non-uniform preservation model, analysed using different calibration approaches (arbitrary maxima at 110 and 150%, stratigraphic and phylogenetic bracketing). Plots are shown for one analysis of a single dataset, with the width of the 95% HPDs plotted against the posterior means. Results are shown for the priors (grey points) and posteriors obtained based on the analysis of one locus and 20 loci. The regression line is shown for the case of 20 loci.

In an infinite-sites plot, posterior interval widths are plotted against the posterior means. As the amount of sequence data approaches infinity, the points will fall on a straight line and the remaining uncertainty in the posterior will be attributed to uncertainty in the calibrations, which imposes a theoretical limit on the precision that can be achieved [3,5]. This pattern can be observed in the infinite-sites plots generated from the simulated data, shown for the prior and posterior results for one and 20 loci (figure 5; electronic supplementary material, figures S6–S8)—these plots show that interval width decreases with more sequence data across all preservation scenarios and calibration methods, and that precision is approaching its theoretical limit (as *R*^2^ = 1); however, note the difference between the slopes for 10 versus 20 loci is small (electronic supplementary material, figures S9–S12). The gradient of the infinite-sites plots is also informative about the degree of uncertainty in the results: a higher gradient corresponds to greater uncertainty. When fossil sampling was low, increased molecular sampling decreased the gradient, but the slope of the line remained steep. The best results were always found at the highest levels of fossil and molecular sampling.

## 4. Discussion

### (a) The impact of non-uniform and variable fossil sampling

Mechanistic models of fossil preservation and molecular evolution are an effective approach to evaluating the impact of fossil sampling and the performance of competing approaches to calibration. The methods we evaluated (stratigraphic bracketing, phylogenetic bracketing and arbitrary maxima) are heuristic and none are demonstrably superior across all scenarios (figures 3 and 4). The success of each approach was dependent on (i) preservation model, (ii) sampling intensity, (iii) tree shape and (iv) the parameters used to construct the calibration density, all of which affect the proximity of first appearances to the true divergence times. These approaches are therefore only reliable insofar as the relationship between these variables can be specified accurately.

Establishing reliable estimates of fossil record completeness is challenging because (i) the mechanisms of diversification and preservation are poorly understood; (ii) the variables that affect the distribution of species and fossils are numerous and complex, and non-uniform across time, space and taxa [1]; and (iii) even naive (e.g. uniform) estimates of sampling require comprehensive databases of fossil occurrences. However, empirical estimates of fossil record completeness do reflect our qualitative perception of variable preservation and sampling rates. For example, the highest estimates of genus preservation probability are obtained for groups of mineralized shallow marine invertebrates [24]. Obtaining higher-resolution, non-uniform per interval estimates of sampling is more challenging—these parameters are unavailable for most, especially poorly preserved, clades due to a paucity of data or lack of reliable methods. Wagner & Marcot [25] developed a novel strategy that explicitly models non-uniform temporal and spatial sampling. Taking advantage of public databases of fossil occurrences available for mammal species, the authors used this approach to estimate 0.0004–0.15 per Myr sampling rates among Cenozoic mammals (equivalent to *p* *=* 0.001–0.3 per interval in this study).

We modelled sampling intensities to reflect a broad range of preservation scenarios, from exceptional (*s*, PA = 1.0) to poor (*s*, PA = 0.001–0.1) preservation. Exceptional preservation is a spatio-temporally unrealistic expectation, but was considered here to explore an ideal. Our simulations demonstrate that at this level of sampling, in general, the results tend to be both more accurate and precise, although the results can still be poor (figure 3). In reality, however, sampling rates for most groups will be closer to the other end of spectrum. At lower values (*s*, PA = 0.001–0.1), the results tended to be less accurate and precise (figures 3 and 4; electronic supplementary material, figure S5). Furthermore, when calibrations are imprecise, the results were more sensitive to the parameters used to specify the calibration densities. At low sampling rates, alternative sources of evidence may be valuable for establishing more precise constraints.

### (b) The impact of tree shape

The impact of tree shape on divergence times has hardly been considered [31]. Most empirical phylogenies exhibit some degree of imbalance that can be attributed to the underlying diversification process and/or non-uniform taxon sampling [32]. We highlight two issues that are created by tree imbalance. First, imbalance leads to greater disparity between divergence times and first appearances when fossil sampling is low and non-uniform. Second, imbalance increases the number of nested nodes and the potential for interaction among overlapping calibrations [11,12]. The results of our simulations suggest both factors can impact divergence estimates: fossil preservation and tree imbalance create a greater disparity between first appearances and divergence times for the unbalanced tree; the impact of truncation creates a disparity between the relative interval width of the specified versus effective priors for the unbalanced tree (electronic supplementary material, tables S1–S4). Importantly, these patterns are also reflected in the posteriors—tree shape led to variable results among different approaches to calibration under equivalent fossil preservation scenarios (figures 3 and 4). This highlights the importance of examining the performance of the specified and effective priors, not merely the posteriors. These results also demonstrate the importance of considering factors affecting divergence time estimation in the context of incomplete, non-uniform fossil preservation.

### (c) The relative impact of fossil and sequence sampling

Empirical calibrations are invariably associated with significant uncertainty [6,14] and, practically, molecular dating serves to minimize this uncertainty. Indeed, genome-scale datasets are thought to improve both the accuracy and precision of molecular divergence times [17–21]. However, accurate posteriors can only be obtained if the calibrations are also (approximately) accurate [3]. The results of our analyses indicate that the addition of more sequence data increases both the accuracy and precision of molecular divergence times (figure 3), but we illustrate the diminishing effects of adding more sequence data. We show that the performance of the priors will be the main driver of accuracy and precision in the posteriors.

Our mechanistic models of fossil preservation and molecular evolution (comparable to the mammalian nuclear genome, in terms of substitution rate) demonstrate that fossil sampling exerts a large influence on the overall precision that can be obtained using the molecular clock (figures 3–5). In empirical studies, there may be several important reasons to collect more sequence data (e.g. to account for among-lineage rate variation [29] or variable coalescence times among loci [33]). However, our results demonstrate that, ultimately, to obtain both accurate and precise estimates of divergence times, the temporal constraints on divergence times must also be accurate and precise. We also demonstrate that this can be achieved with increased fossil sampling, but though both pursuits are worthwhile, for many empirical datasets, the acquisition of sequence data may be more straightforward than collecting more fossil data.

### (d) Approximating the posterior distribution of ages

In Bayesian divergence time estimation, the ages sampled using MCMC methods are intended to approximate the posterior distribution. It is convenient to describe divergence estimates using the mean along with the 95% HPDs of the posterior distribution. As the distribution of molecular divergence estimates is often asymmetric, the median and other summary statistics have been proposed as alternatives to the mean to provide a better approximation of the results [34,35]. This relies on the assumption that molecular divergence estimates will converge on the truth. However, our simulations demonstrate that referring to age estimates on the basis of a single value can be misleading, especially when fossil sampling is low and there is a great deal of uncertainty in the calibrations (figure 2). The mean and median sometimes provide an extremely poor approximation of the true age. Furthermore, a single value fails to reflect the uncertainty associated with divergence times and hence the precision with which a node age is known based on available evidence. The posterior distribution better reflects the uncertainty associated with both fossil and molecular sampling, and the 95% HPD is more likely (though not guaranteed) to encompass the true age, especially when that uncertainty is large (figure 2). Reporting divergence times using a single value perpetuates an illusion of precision [36], and adopting mean or median values in downstream analyses [37] can further propagate associated errors. We cannot know most evolutionary divergence times to within 1% of clade age, especially the evolution of clades that occurred over time scales of tens of millions to billions of years. At these time scales, there is invariably a great deal of uncertainty in the calibrations. In any molecular dating study, the results should be interpreted on the basis of the Bayesian credibility intervals, or the 95% HPDs. Though more reliable, the credibility intervals impose a limit on the temporal resolution at which we are able to answer biologically meaningful questions. If the degree of precision required to test an evolutionary hypothesis cannot be achieved, then those questions may be beyond the scope of scientific enquiry.

In a conventional statistical estimation problem, the point estimate can be assessed by its bias (the difference between the expected estimate from the true parameter value) and variance, with the expectation that the point estimate will converge to the true value and the variance will go to zero when the amount of data approaches infinity. The confidence or credibility interval for the parameter is expected to have the correct coverage: that is, the 95% interval should include the true value in 95% of the datasets. Bayesian divergence time estimation is unconventional in that the sampling error or variance in the point estimate does not converge to zero, so that uncertainty persists even if an infinite amount of sequence data is available, due to the fact that time and rate are confounded in the comparison of molecular sequences [3–5]. Judged by the statistical criteria of bias, variance and coverage, Bayesian molecular clock dating, as evaluated in this study, must be considered to produce very poor estimates. The point estimates had wide credibility intervals, often so wide that the estimates would be effectively uninformative in testing evolutionary hypotheses. Similarly, the credibility intervals rarely had coverage greater than 95%. We suggest that this poor performance partly reflects the difficulty posited by the confounding effect of time and rate. In several ways, our analysis reflects the best-case scenario—the topology and the age and placement of fossils are known without error, and with the exception of the tree and calibration priors, the priors and models match those used to generate the data—so empirical analyses are expected to be even more challenging.

The methods for constructing calibrations evaluated here are simple and heuristic, and produce reasonable results (in terms of accuracy and precision) when fossil sampling is uniform and high—a scenario rarely encountered in reality. Improving the molecular divergence estimates for most clades will require focusing on calibration approaches that use more fossil data, and have the potential to incorporate mechanistic models of fossil preservation and recovery [38–40]. Furthermore, as sampling and diversity are linked, we welcome the development of models that allow for the co-estimation of divergence, diversification and sampling parameters, or enable the estimation and specification of rates during independent intervals [8,41,42]. We suggest that accumulation of suitable fossil data (both fossil presence/absence data and morphological measurements) and the development of advanced statistical inference methods for their analysis will lead to better fossil calibrations, which will eventually improve our molecular clock estimates of divergence times.

## 5. Conclusion

The accuracy of molecular estimates of divergence times cannot simply be improved with the addition of more sequence data alone. The accuracy and precision of divergence times are also driven by the accuracy and precision of the calibrations. Improving estimates of evolutionary time will therefore greatly benefit from further development of methods that use more fossil data, and can account for non-uniform preservation and sampling. However, all available methods require an appreciably large amount of high quality fossil data to obtain precise divergence time estimates, which is unavailable for many groups. Ultimately, however, this is a worthwhile pursuit, because for groups that have a sparse fossil record, the molecular clock provides our only means of establishing an evolutionary time scale. In cases where fossil sampling cannot be improved, modelling alternative parameters, such as diversification rates, may be especially beneficial. Otherwise, calibration strategy and gene sampling intensity should be guided by calibration precision and fossil sampling intensity. Imprecise calibrations can only deliver imprecise divergence time estimates. Finally, we highlight the importance of reporting divergence times on the basis of the 95% credible interval to represent the posterior, rather than a more precise proxy, such as the commonly used mean or median age, as these are invariably a poor approximation of the true age. Together, our results demonstrate that the incomplete and non-uniform nature of the fossil record should be an integral component of developing and testing molecular dating methods.

## Supplementary Material

Supplementary methods and figures

## Data accessibility

Code used to perform all analyses is available online [30].
