# Supplementary material for: Testing the molecular clock using mechanistic models of fossil preservation and molecular evolution
Source: Proc Biol Sci. 2017 Jun 21;284(1857):20170227. doi: 10.1098/rspb.2017.0227 (PMC5489717; doi:10.1098/rspb.2017.0227)
Supplement: Supplementary methods and figures [file rspb20170227supp1.pdf]

Supplementary material and methods

(a) Simulation of fossil occurrence and sequence data

Stratigraphic occurrences of fossils were simulated for two trees of 16 extant taxa, one balanced and one unbalanced, under both uniform and non-uniform models of preservation (figure 1). We preferred the use of fixed topologies to random trees generated from the birth-death process, as fixed trees make the interpretation of the simulation results much easier. The age of the root is fixed at 100 Ma, which is defined as one time unit. The time period between the present and the age of the root was subdivided into 50 stratigraphic intervals of equal length (2 Myr). During each interval, each extant lineage was collected with a probability that is the product of sampling probability p and the fraction of the interval during which the lineage is extant. The sampling probability p here reflects the joint effects of fossil preservation potential and sampling intensity, effects that are indistinguishable in such a model. Under the uniform model of preservation p is simply equal to the specified sampling intensity, s. We used four values: s = 0.001, 0.01, 0.1, and 1, to reflect the perceived completeness of the fossil record [1, 2].

To simulate non-uniform occurrence data we used a three-parameter Gaussian model of preservation [3, 4], which uses water depth as a proxy for fossil preservation or sampling potential in the marine stratigraphic record. The sampling probability is given by

$$p = \mathrm{PA} \times e^{-\frac{1}{2\mathrm{DT}^2}(d-\mathrm{PD})^2}, \tag{1}$$

where d is the current water depth, PD is preferred depth, DT is depth tolerance and PA is peak abundance of the species. PD and DT are the mean and standard deviation of the distribution, respectively. Water depth (d) was simulated using a simple sine wave function

$$d(t) = 2\sin\{2\pi(t-\tfrac{1}{4})\}. \tag{2}$$

This emulates two successive regression and transgression events over the time interval (0 – 100 Myr), with a relative maximum and minimum depth of 2 and –2, respectively. The preservation potential was assumed to be constant across contemporary lineages of the tree (see figure 1). We used four values of peak abundance, PA = 0.001, 0.01, 0.1, and 1, with PD = 1 and DT = 1 fixed. Example datasets of sampled fossils on both balanced and unbalanced trees are shown in figure 1.

Sequence alignments were simulated using the program evolver in PAML 4.8 [5]. We generated data with L = 1, 2, 10 or 20 loci, with 1000 base pairs at each locus. For each locus i, an overall mean rate μ_i was sampled from a gamma distribution, $G(2, 2)$, with the mean 1

substitution/site/unit time or 10^{-8} substitutions/site/year. Then independent rates for branches on the tree were sampled from a lognormal distribution with mean rate μ_i and standard deviation of the log rate $\sigma = 0.1$ (e.g. [6]). This is the independent-rates model and allows variable rates both among multiple loci and among branches at each locus. Branch lengths, in the expected number of substitutions per site, were calculated as the product of the time duration of the branch and the rate. Given the tree topology and branch lengths, the HKY85+Γ5 substitution model was used to 'evolve' the sequences along the tree, with equal base frequencies, a transition to transversion rate ratio $\kappa = 5$, and the gamma shape parameter $\alpha = 0.25$ for rate heterogeneity across sites. The number of replicate datasets is 100, and each replicate dataset consists of L loci.

(b) Minimum and maximum constraints on divergence times

The simulated fossil occurrence data was used to establish minimum (t_L) and maximum (t_U) constraints on ages of nodes on the tree, which were used as calibrations in Bayesian estimation of divergence times (figure S1). In all cases we assumed no topological uncertainty in the placement of fossils or the relationships among modern terminals. Minimum constraints were based on first appearances, using the youngest age of the time bin from which the fossil was recorded. Three approaches were used to establish maximum constraints. First, we used a stratigraphic bracketing based approach, which uses the density of fossil occurrences over a given interval to estimate 95% confidence intervals on stratigraphic ranges ([7], equation 14 – modified from [8]),

$$t_U = \frac{t_L}{bH\sqrt{0.05}}, \tag{3}$$

where b is the number of lineages with a fossil record, and H is the number of fossil localities. For a given node in each tree, H was calculated using the combined occurrence data of the descendent branches that may be ancestral to younger nodes in the phylogeny. In equation 3, t_L is given by the age of the oldest fossil. Second, phylogenetic bracketing was used to emulate best-practice approaches of establishing calibrations (e.g. [9-11]). Maxima were established based on the oldest age interpretation of the minimum constraints of ancestral clades. For a given node j, if the immediate ancestor did not have a minimum constraint older than the minimum of j, the next nearest ancestor with a minimum older than j was used to inform the maximum of j. Maxima were not established for nodes that did not have a minimum constraint. This approach will not produce a maximum constraint for the root of the tree. Third, we generated the maximum bounds to be 110, 125, 150 and 175% the age of the minimum constraints. In all cases, the age of the fossil used to inform the maximum constraints was equal to the maximum age of the interval from which the fossil was sampled.

(c) Calibration densities

We implemented three fossil calibration strategies in the molecular clock dating analysis using MCMCTREE. First, we used the minimum and maximum fossil-constraints obtained using stratigraphic and phylogenetic bracketing to generate soft-uniform bounds [12]. We use sharp minimum and soft maximum bounds, with tail probability $p_L = 0.1\%$ on the left and $p_U = 2.5\%$ on the right. Second, we used the skew-*t* distribution and specified the parameters by attempting to match the minimum and maximum bounds for each calibration node with the 0.1% and 97.5% percentiles of the distribution, respectively. The minimum and arbitrary maximum bounds were also implemented using the skew-*t* distribution using this approach. All calibration strategies are illustrated in figure S1.

We always applied a soft uniform calibration at the root of the tree, with tail probabilities p_L = 0.1% on the left and p_U= 2.5% on the right. When there was insufficient data to inform the maximum constraint at the root, this was set to twice the true age for the root (200 Ma). If no fossils were sampled at all, the root age is assigned a uniform distribution over the interval $U(0, 2)$.

(d) Molecular clock analysis and MCMC Settings

The MCMCTREE program [13] was used to date species divergences, using the sequence alignments at L loci. The proportion of calibrated nodes on the tree varied from 0 to 1 among the trees/calibration strategies: in some datasets no fossils were sampled and no fossil calibrations were generated, while in some other datasets every node had a calibration. The prior on times for the non-calibration nodes was generated from the birth-death-sampling process, with parameters $\lambda = 1, \mu = 1$ and $\varrho = 0$. These parameter values generate a uniform density kernel [13]. The likelihood for the sequence alignments was calculated using the approximate method of dos Reis & Yang [14]. The program baseml was used to calculate the maximum likelihood estimates of branch lengths and the Hessian matrix under the HKY+Γ_5 substitution model, as well as substitution parameters $\varkappa$ and α in the model.

In the analysis of the multi-loci sequence data, we used the gamma-Dirichlet prior [15] on the rates for loci (μ_i), implemented in MCMCTREE. A gamma prior is assigned on the average rate among loci, $\bar{\mu} \sim G(2, 2)$, with mean 1 (or 10^{-8} substitutions/site/year) and standard deviation 0.7, and a uniform Dirichlet distribution is used to partition the total rate for all loci into rates for the L loci (μ_i,). Given the rate μ_i for locus i, the branch rates at the locus are assigned independent lognormal distributions with the variance parameter σ_i^2. This is the

independent rates model. Similar to the overall locus rates μ_i, the variance parameters (σ_i^2) are assigned a gamma-Dirichlet prior, with the average of σ_i^2 having a gamma prior $G(1, 10)$, with mean 0.1 and standard deviation 0.1.

We note that at the lowest level of fossil recovery ($PA = 0.001$) sampling events are rare and so a large proportion of tree replicates will have no internal fossil-based constraints. Time estimates are then strongly influenced by the loose constraint applied on the age of the root node.

A subset of the datasets were used to assess convergence and select MCMC settings for different alignment lengths, by examining the differences between independent runs and ensuring ESS values were >200. After a burn-in of 4000 iterations, 20 000 samples were taken, sampling every n iterations, where n was determined independently for each alignment length. In total we performed 64 000 molecular clock analyses.

(e) Performance measures

We considered the posterior means and medians of divergence times as the point estimates and the 95% highest posterior density credibility intervals (or 95% HPD CIs) as the interval estimates. The coverage probability for any node age is estimated using the proportion of simulated replicates in which the CI included the true age, and this is averaged over all nodes to provide an overall measure. Precision of the interval estimate is measured by the width of the 95% HPD CI, divided by the true node age, and then averaged over all nodes on the tree and over the simulated replicates to provide an overall measure for the method. CI coverage and width can be calculated for both the prior and the posterior. As a wide interval may not be very useful even if it includes the truth, we use the relative root mean square error (RMSE) as a combined measure of both accuracy and precision. This is defined as $\frac{1}{t}\sqrt{\mathrm{MSE}(\hat{t}-t)} = \frac{1}{t}\sqrt{\frac{1}{n}\sum_i(\hat{t}_i - t)^2}$, where t is the true age of a node, and $\hat{t}_i$ is the estimated node age in simulation replicate i. The RMSE is calculated for each node on the tree and then averaged across nodes and across simulated replicates to provide an overall measure.

The complete experimental design is outlined in figure S2. All code used to perform the analysis is available on dryad (doi:10.5061/dryad.5706p) and the models used to simulate fossil data have been made available as part of an R package FossilSim. The development version can be downloaded here: https://github.com/rachelwarnock/fossilsim.

Supplementary material references

1. Foote M, Sepkoski JJ. 1999 Absolute measures of the completeness of the fossil record. *Nature* **398**, 415-417. (doi:10.1038/18872).

2. Wagner PJ, Marcot JD. 2013 Modelling distributions of fossil sampling rates over time, space and taxa: assessment and implications for macroevolutionary studies. *Methods Ecol. Evol.* **4**, 703-713. (doi:10.1111/2041-210x.12088).

3. Holland SM. 1995 The Stratigraphic distribution of fossils. *Paleobiology* **21**, 92-109.

4. Holland SM. 2000 The quality of the fossil record: a sequence stratigraphic perspective. *Paleobiology* **26**, 148-168. (doi:10.1666/0094- 8373(2000)26[148:Tqotfr]2.0.Co;2).

5. Yang Z. 2007 PAML 4: Phylogenetic analysis by maximum likelihood. *Mol. Biol. Evol.* **24**, 1586-1591. (doi:10.1093/molbev/msm088).

6. Brown RP, Yang Z. 2011 Rate variation and estimation of divergence times using strict and relaxed clocks. *BMC Evol. Biol.* **11**. (doi:10.1186/1471-2148-11- 271).

7. Marshall CR. 2008 A simple method for bracketing absolute divergence times on molecular phylogenies using multiple fossil calibration points. *Am. Nat.* **171**, 726- 742. (doi:10.1086/587523).

8. Strauss D, Sadler PM. 1989 Classical confidence-intervals and Bayesian probability estimates for ends of local taxon ranges. *Math. Geol.* **21**, 411-421. (doi:10.1007/Bf00897326).

9. Benton MJ, Donoghue PCJ. 2007 Paleontological evidence to date the tree of life. *Mol. Biol. Evol.* **24**, 26-53. (doi:10.1093/molbev/msl150).

10. Muller J, Reisz RR. 2005 Four well-constrained calibration points from the vertebrate fossil record for molecular clock estimates. *Bioessays* **27**, 1069-1075. (doi:10.1002/bies.20286).

11. Parham JF et al. 2012 Best practices for justifying fossil calibrations. *Syst. Biol.* **61**, 346-359. (doi:10.1093/sysbio/syr107).

12. Yang Z, Rannala B. 2006 Bayesian estimation of species divergence times under a molecular clock using multiple fossil calibrations with soft bounds. *Mol. Biol. Evol.* **23**, 212-226. (doi:10.1093/molbev/msj024).

13. Yang Z, Rannala B. 1997 Bayesian phylogenetic inference using DNA sequences: A Markov Chain Monte Carlo method. *Mol. Biol. Evol.* **14**, 717-724.

14. dos Reis M, Yang Z. 2011 Approximate likelihood calculation on a phylogeny for Bayesian estimation of divergence times. *Mol. Biol. Evol.* **28**, 2161-2172. (doi:10.1093/molbev/msr045).

15. dos Reis M, Zhu TQ, Yang Z. 2014 The impact of the rate prior on Bayesian estimation of divergence times with multiple loci. *Syst. Biol.* **63**, 555-565. (doi:10.1093/sysbio/syu020).

Supplementary figure legends

Figure S1. Example calibrations generated for a given node in the unbalanced tree, using the fossil occurrence data simulated under the non-uniform model of preservation with variable sampling intensities ((a): $PA = 1.0$, (c): $PA = 0.1$). The same focal node is indicated by a star in (a) and (b). The minimum and maximum constraints were generated using stratigraphic bracketing, phylogenetic bracketing and arbitrary maxima (at 110% and 150%). Minimum constraints were always based on the youngest secure age interpretation of first appearances. The fossils used to inform the maximum constraints are highlighted in the trees, including stratigraphic bracketing or phylogenetic bracketing, which uses the oldest secure age of the first appearance of the nearest ancestral clade. The age interpretation of the fossil specimens used to define minimum or maximum constraints are indicated by the dashed grey line. The minimum and maximum constraints were used to construct uniform or skew-*t* prior densities in Bayesian molecular clock analysis, shown right, in (b) and (c). In these plots, the solid grey line indicates the true node age.

Figure S2. Outline of the experimental design, indicating the main stages – data generation (shown in orange), calibration generation (green), molecular clock analysis (blue) and analysis of output (red) – and the parameters used throughout the simulations.

Figure S3. The fully balanced and unbalanced trees, indicating the numbered nodes used to display the distribution of point estimates shown in figure 2 and figure S3.

Figure S4. The posterior means (red or yellow) and medians (blue) of the node ages for four selected nodes (indicated in figure S2) in the 100 replicate datasets are plotted against the true node ages (black triangles) for data simulated using the balanced tree under the non-uniform model of fossil sampling, given low versus high sampling intensities ($PA = 0.001$ or 1.0). The methods of analysis include arbitrary maxima (at 110% and 150%), stratigraphic bracketing, and phylogenetic bracketing, using skew-*t* calibration densities.

Figure S5. Average precision for the ages in datasets simulated under different conditions analyzed using different calibration approaches. Sampling intensity is *PA* under the non-uniform model of fossil preservation or *s* in the uniform sampling model. The colored lines show the results obtained for different calibration approaches: arbitrary maxima, stratigraphic bracketing and phylogenetic bracketing. Each point represents the normalized precision averaged over the 15 nodes on the tree (figure 1) and over the 100 replicate datasets.

Figure S6-S8. Infinite-sites plots for data simulated on the unbalanced tree under the uniform preservation model (S5) and the balanced tree under the non-uniform (S6) and uniform (S7) models and analyzed using different calibration approaches (arbitrary maxima at 110% and 150%, stratigraphic bracketing and phylogenetic bracketing). The infinite-sites plot is shown for one analysis of a single dataset, with the widths of the 95% HPD intervals for the 15 node ages plotted against their posterior means. The results are shown for the priors (grey points) and the posteriors obtained based on the analysis of 1 locus and 20 loci. The regression line is shown for the analysis using 20 loci.

Figure S9-S12. Infinite-sites plots for data simulated on the unbalanced tree under the non-uniform (S8) and uniform (S9) preservation models and the balanced tree under the non-uniform (S10) and uniform (S11) models and analyzed using different calibration approaches (arbitrary maxima at 110% and 150%, stratigraphic bracketing and phylogenetic bracketing). The infinite-sites plot is shown for one analysis of a single dataset, with the widths of the 95% HPD intervals for the 15 node ages plotted against their posterior means. The results are shown for the priors (grey points) and the posteriors obtained based on the analysis of 1 locus and 10 loci. The regression line is shown for the analysis using 10 loci.

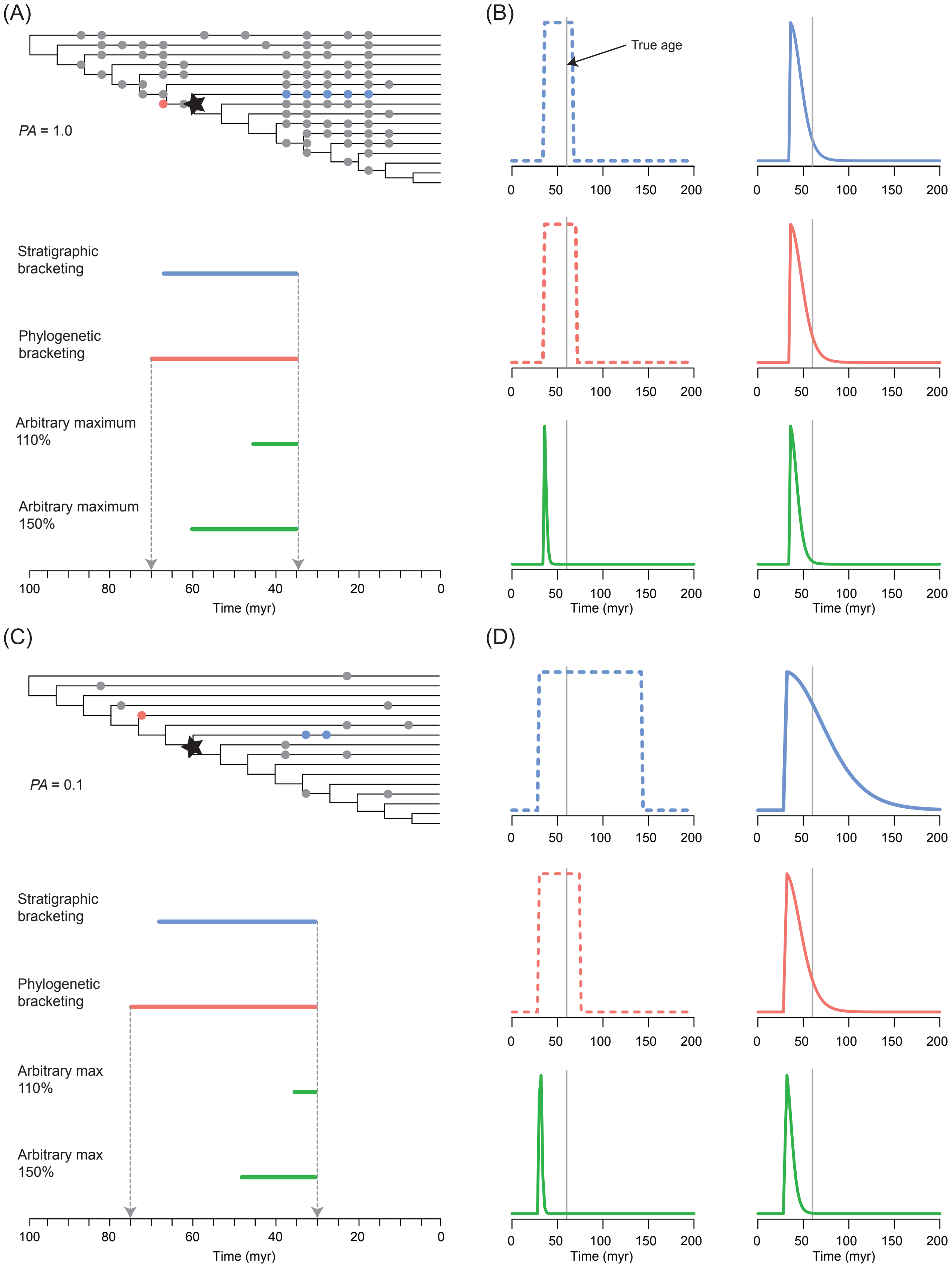

(A)
PA = 1.0
Stratigraphic bracketing
Phylogenetic bracketing
Arbitrary maximum 110%
Arbitrary maximum 150%
100 80 60 40 20 0
Time (myr)
(B)
True age
0 50 100 150 200
Time (myr)
(C)
PA = 0.1
Stratigraphic bracketing
Phylogenetic bracketing
Arbitrary max 110%
Arbitrary max 150%
100 80 60 40 20 0
Time (myr)
(D)
0 50 100 150 200
Time (myr)

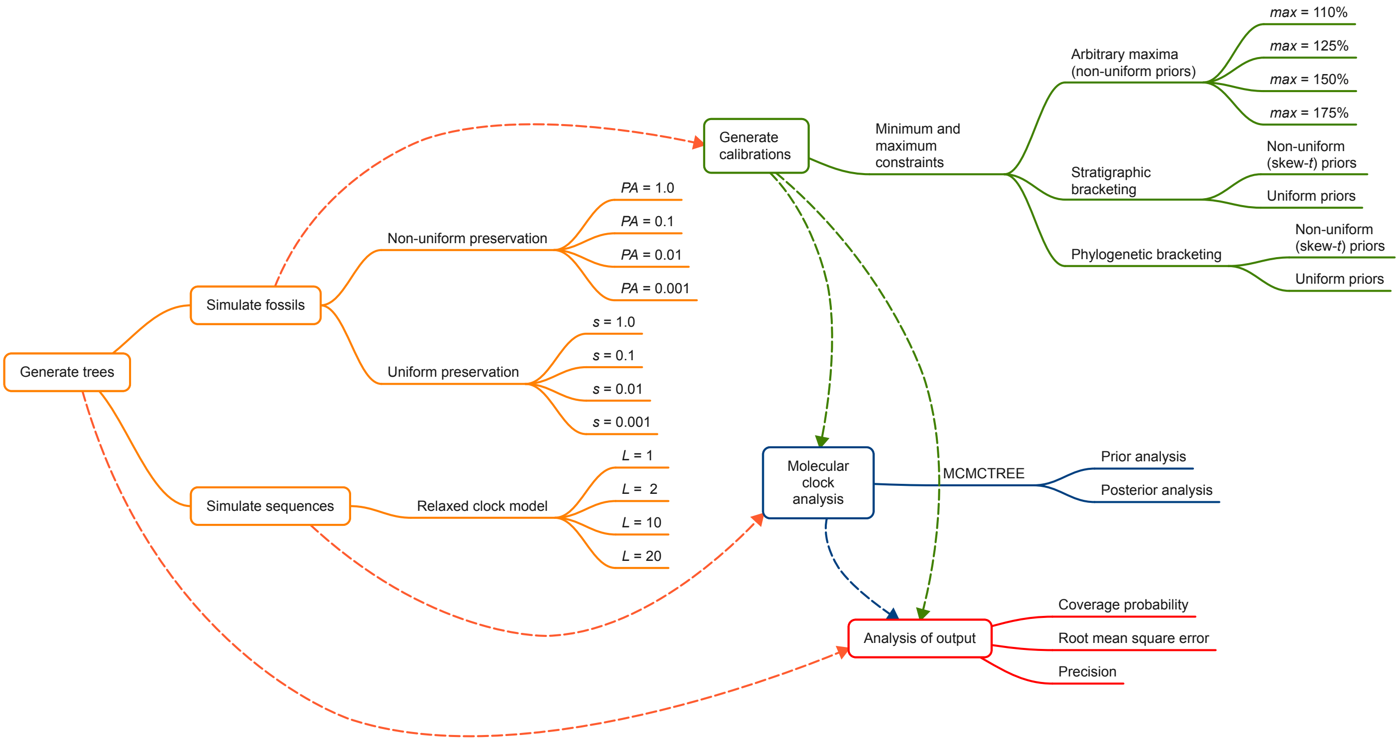

Generate trees
Simulate fossils
Non-uniform preservation
PA = 1.0
PA = 0.1
PA = 0.01
PA = 0.001
Uniform preservation
s = 1.0
s = 0.1
s = 0.01
s = 0.001
Simulate sequences
Relaxed clock model
L = 1
L = 2
L = 10
L = 20
Generate calibrations
Minimum and maximum constraints
Arbitrary maxima (non-uniform priors)
max = 110%
max = 125%
max = 150%
max = 175%
Stratigraphic bracketing
Non-uniform (skew-t) priors
Uniform priors
Phylogenetic bracketing
Non-uniform (skew-t) priors
Uniform priors
Molecular clock analysis
MCMCTREE
Prior analysis
Posterior analysis
Analysis of output
Coverage probability
Root mean square error
Precision

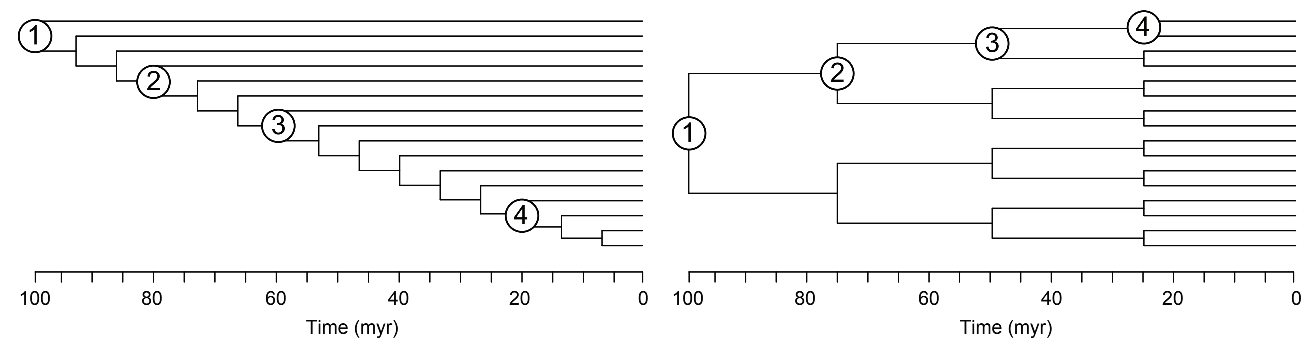
1
2
3
4
100
80
60
40
20
0
Time (myr)
1
2
3
4
100
80
60
40
20
0
Time (myr)

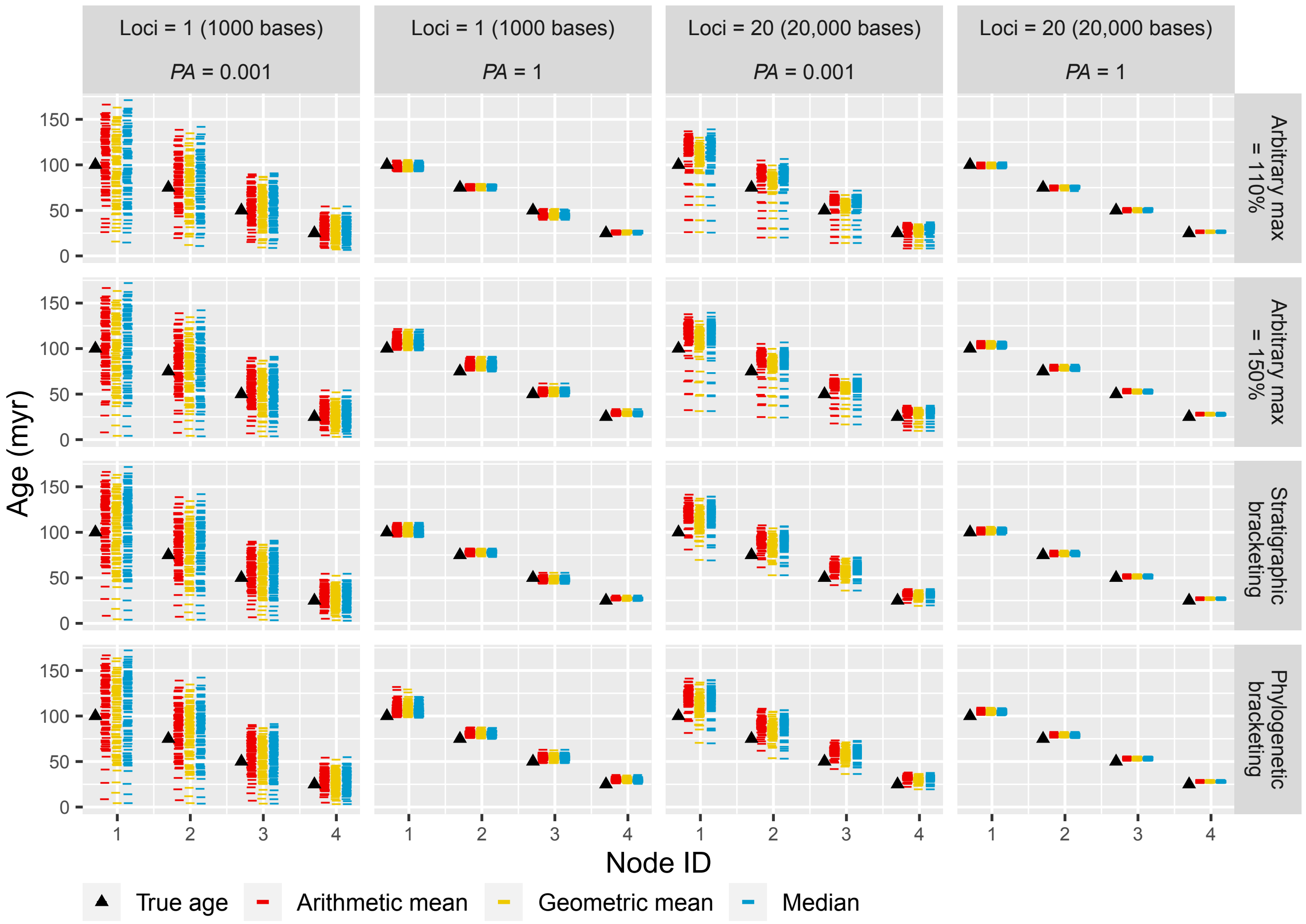
Loci = 1 (1000 bases)
PA = 0.001
Loci = 1 (1000 bases)
PA = 1
Loci = 20 (20,000 bases)
PA = 0.001
Loci = 20 (20,000 bases)
PA = 1
Arbitrary max = 110%
Arbitrary max = 150%
Stratigraphic bracketing
Phylogenetic bracketing
Age (myr)
150
100
50
0
1
2
3
4
Node ID
True age
Arithmetic mean
Geometric mean
Median

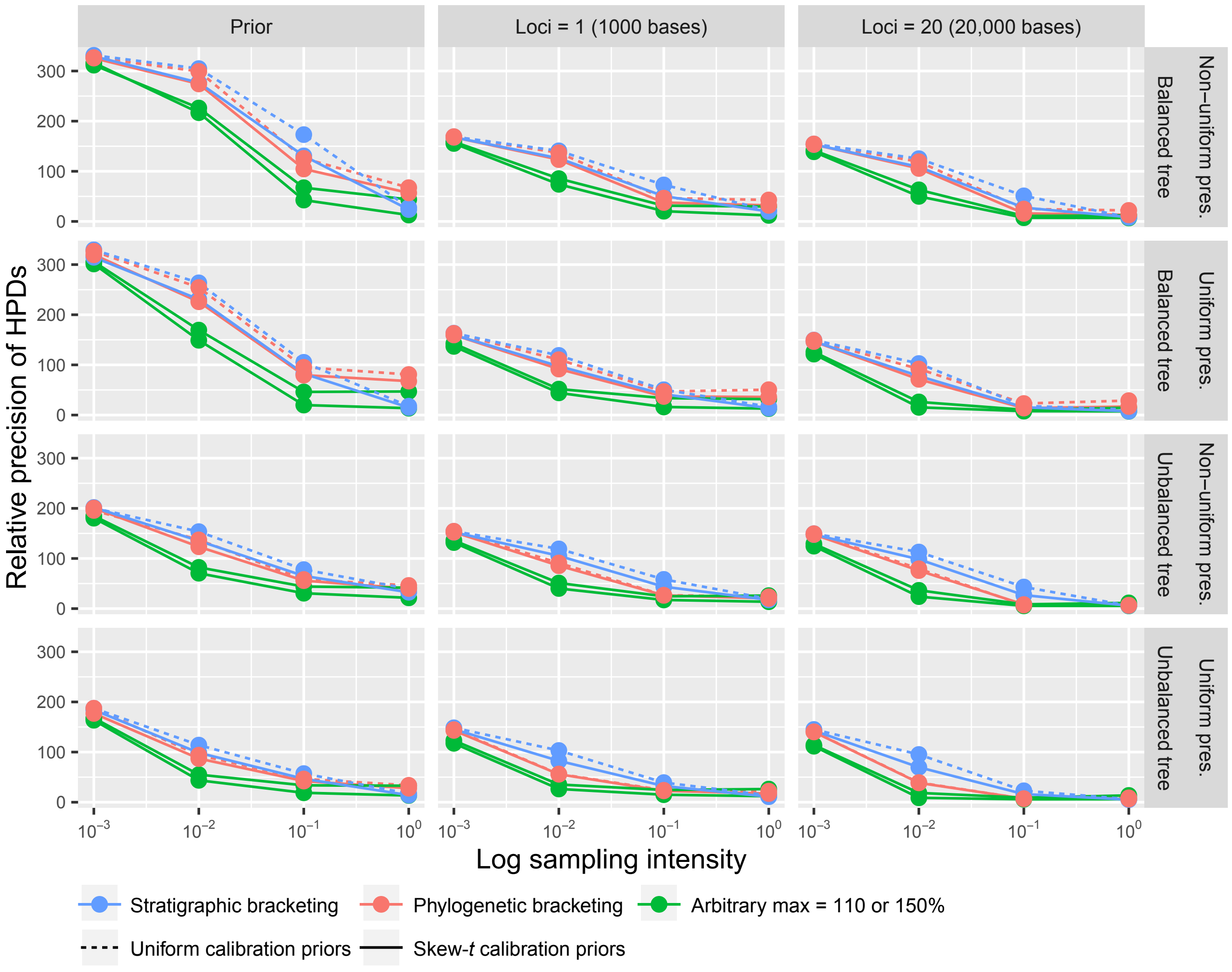

Prior
Loci = 1 (1000 bases)
Loci = 20 (20,000 bases)
Non–uniform pres.
Balanced tree
Uniform pres.
Balanced tree
Non–uniform pres.
Unbalanced tree
Uniform pres.
Unbalanced tree
Relative precision of HPDs
0
100
200
300
10^{-3}
10^{-2}
10^{-1}
10^{0}
Log sampling intensity
Stratigraphic bracketing
Phylogenetic bracketing
Arbitrary max = 110 or 150%
Uniform calibration priors
Skew-*t* calibration priors

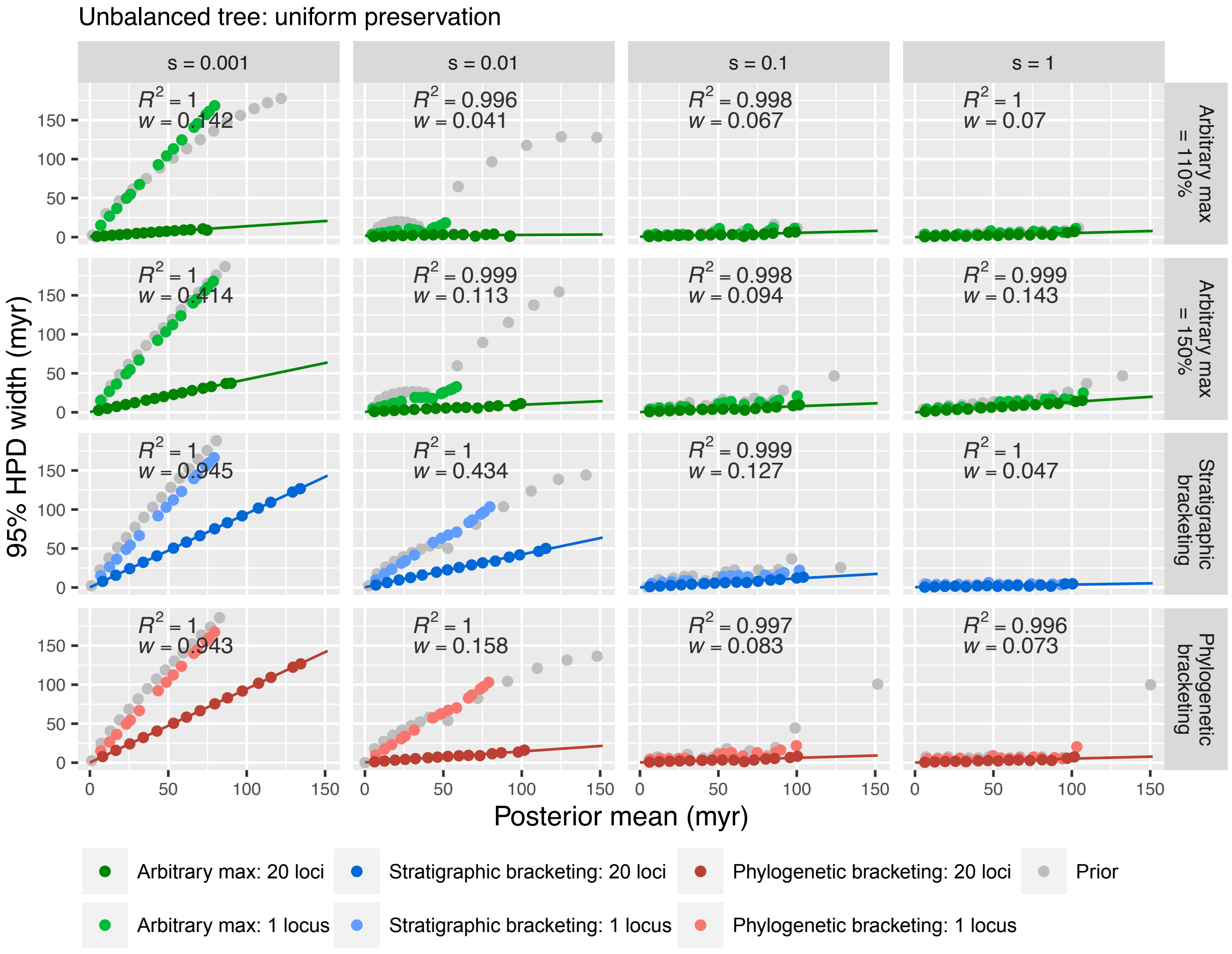

Unbalanced tree: uniform preservation
s = 0.001
s = 0.01
s = 0.1
s = 1
Arbitrary max = 110%
Arbitrary max = 150%
Stratigraphic bracketing
Phylogenetic bracketing
R2 = 1 w = 0.142
R2 = 0.996 w = 0.041
R2 = 0.998 w = 0.067
R2 = 1 w = 0.07
R2 = 1 w = 0.414
R2 = 0.999 w = 0.113
R2 = 0.998 w = 0.094
R2 = 0.999 w = 0.143
R2 = 1 w = 0.945
R2 = 1 w = 0.434
R2 = 0.999 w = 0.127
R2 = 1 w = 0.047
R2 = 1 w = 0.943
R2 = 1 w = 0.158
R2 = 0.997 w = 0.083
R2 = 0.996 w = 0.073
95% HPD width (myr)
Posterior mean (myr)
0
50
100
150
Arbitrary max: 20 loci
Stratigraphic bracketing: 20 loci
Phylogenetic bracketing: 20 loci
Prior
Arbitrary max: 1 locus
Stratigraphic bracketing: 1 locus
Phylogenetic bracketing: 1 locus

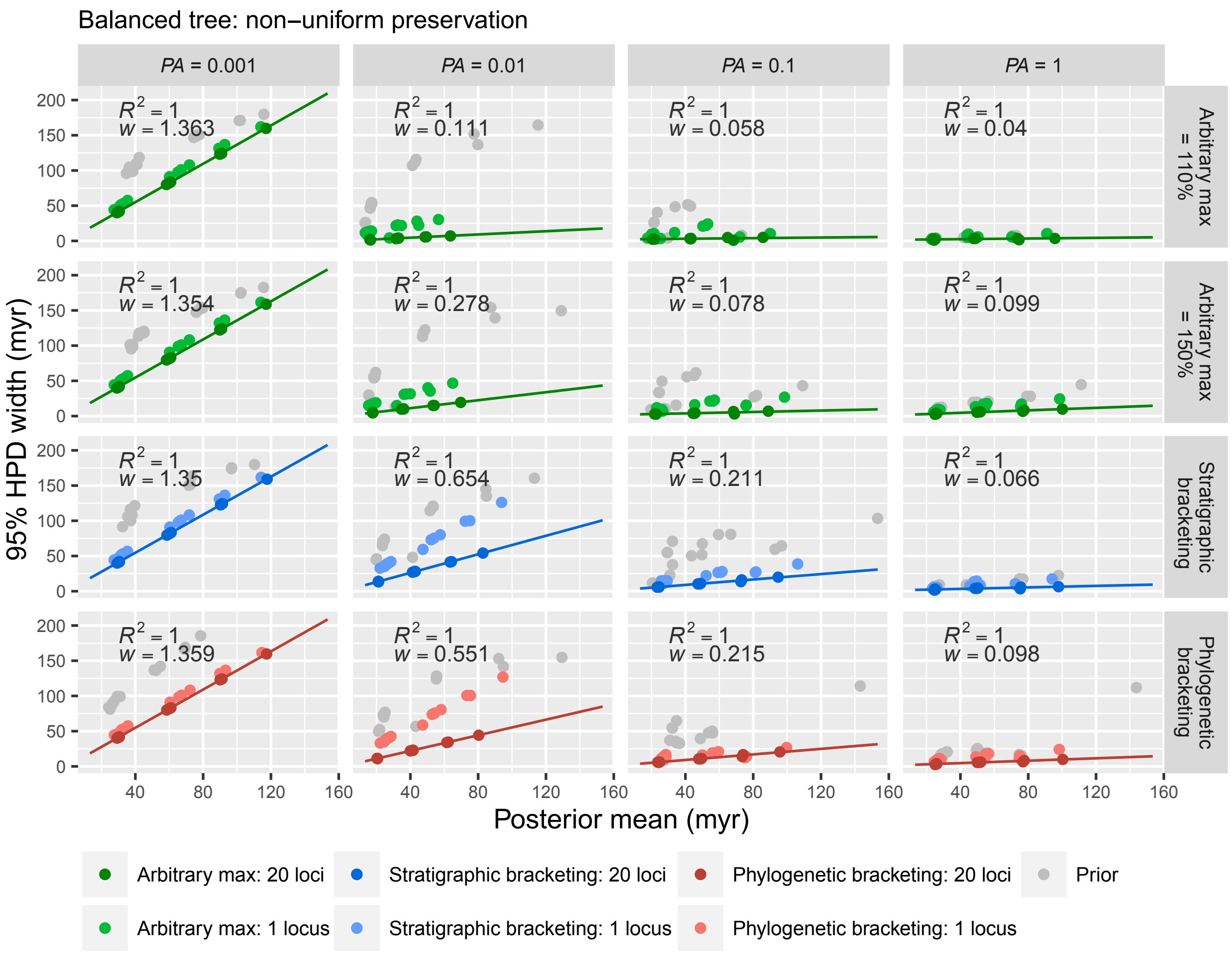

Balanced tree: non−uniform preservation
PA = 0.001
PA = 0.01
PA = 0.1
PA = 1
Arbitrary max = 110%
Arbitrary max = 150%
Stratigraphic bracketing
Phylogenetic bracketing
R2 = 1
w = 1.363
w = 0.111
w = 0.058
w = 0.04
w = 1.354
w = 0.278
w = 0.078
w = 0.099
w = 1.35
w = 0.654
w = 0.211
w = 0.066
w = 1.359
w = 0.551
w = 0.215
w = 0.098
95% HPD width (myr)
Posterior mean (myr)
Arbitrary max: 20 loci
Stratigraphic bracketing: 20 loci
Phylogenetic bracketing: 20 loci
Prior
Arbitrary max: 1 locus
Stratigraphic bracketing: 1 locus
Phylogenetic bracketing: 1 locus

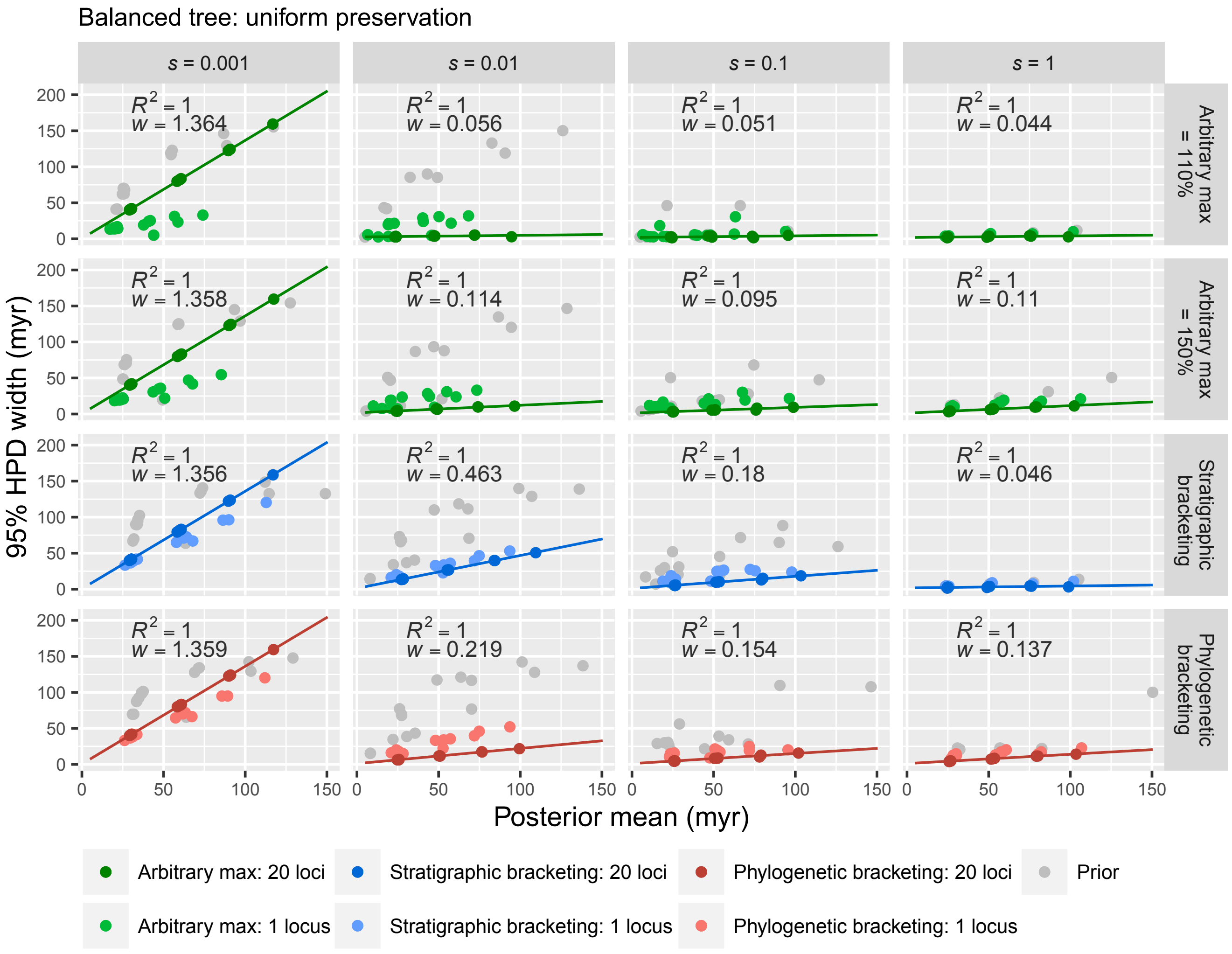
Balanced tree: uniform preservation
s = 0.001
s = 0.01
s = 0.1
s = 1
Arbitrary max = 110%
Arbitrary max = 150%
Stratigraphic bracketing
Phylogenetic bracketing
R2 = 1 w = 1.364
R2 = 1 w = 0.056
R2 = 1 w = 0.051
R2 = 1 w = 0.044
R2 = 1 w = 1.358
R2 = 1 w = 0.114
R2 = 1 w = 0.095
R2 = 1 w = 0.11
R2 = 1 w = 1.356
R2 = 1 w = 0.463
R2 = 1 w = 0.18
R2 = 1 w = 0.046
R2 = 1 w = 1.359
R2 = 1 w = 0.219
R2 = 1 w = 0.154
R2 = 1 w = 0.137
95% HPD width (myr)
Posterior mean (myr)
Arbitrary max: 20 loci
Stratigraphic bracketing: 20 loci
Phylogenetic bracketing: 20 loci
Prior
Arbitrary max: 1 locus
Stratigraphic bracketing: 1 locus
Phylogenetic bracketing: 1 locus

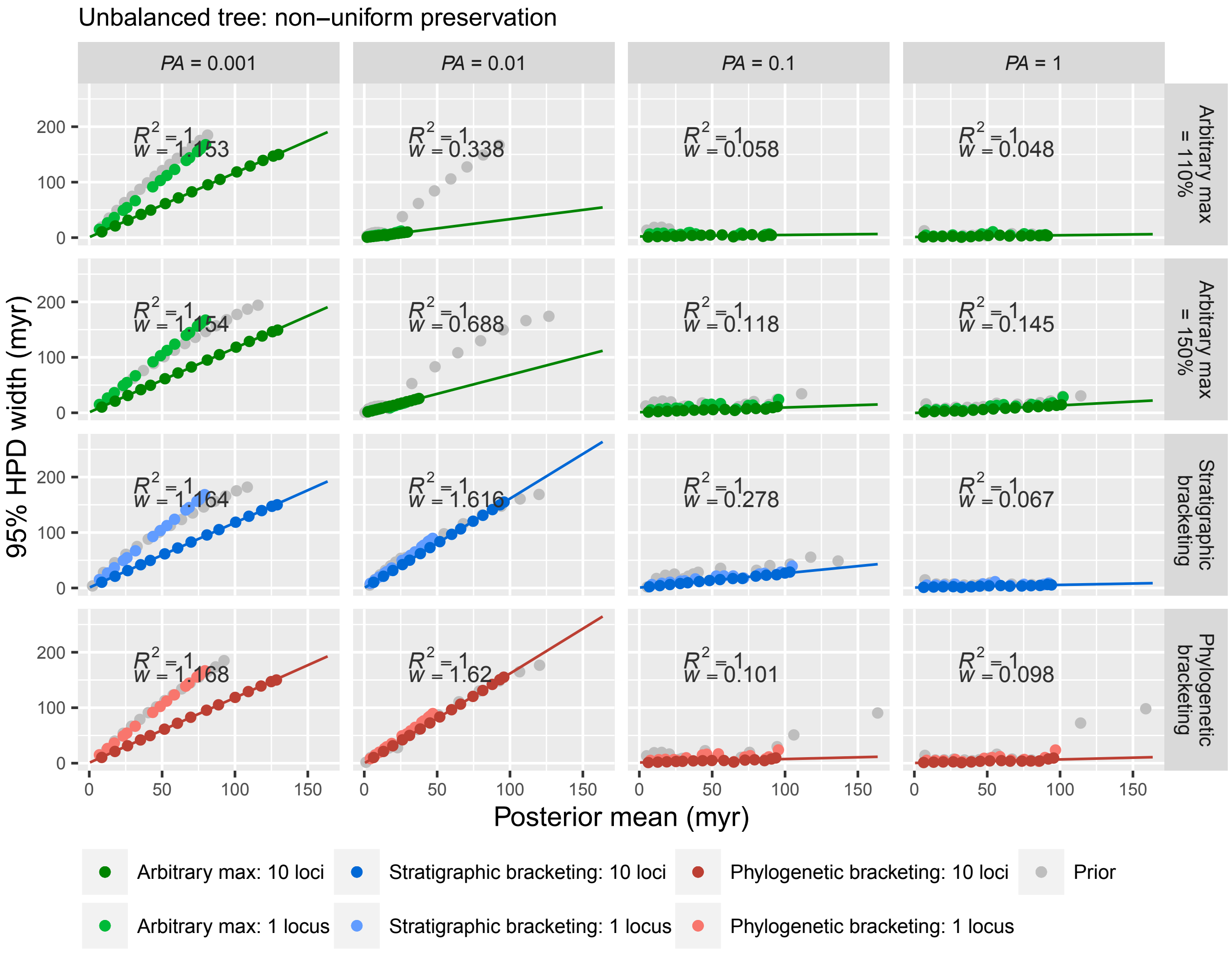

Unbalanced tree: non−uniform preservation
PA = 0.001
PA = 0.01
PA = 0.1
PA = 1
Arbitrary max = 110%
Arbitrary max = 150%
Stratigraphic bracketing
Phylogenetic bracketing
R² = 1
w = 1.153
w = 0.338
w = 0.058
w = 0.048
w = 1.154
w = 0.688
w = 0.118
w = 0.145
w = 1.164
w = 1.616
w = 0.278
w = 0.067
w = 1.168
w = 1.62
w = 0.101
w = 0.098
95% HPD width (myr)
Posterior mean (myr)
0
50
100
150
200
Arbitrary max: 10 loci
Arbitrary max: 1 locus
Stratigraphic bracketing: 10 loci
Stratigraphic bracketing: 1 locus
Phylogenetic bracketing: 10 loci
Phylogenetic bracketing: 1 locus
Prior

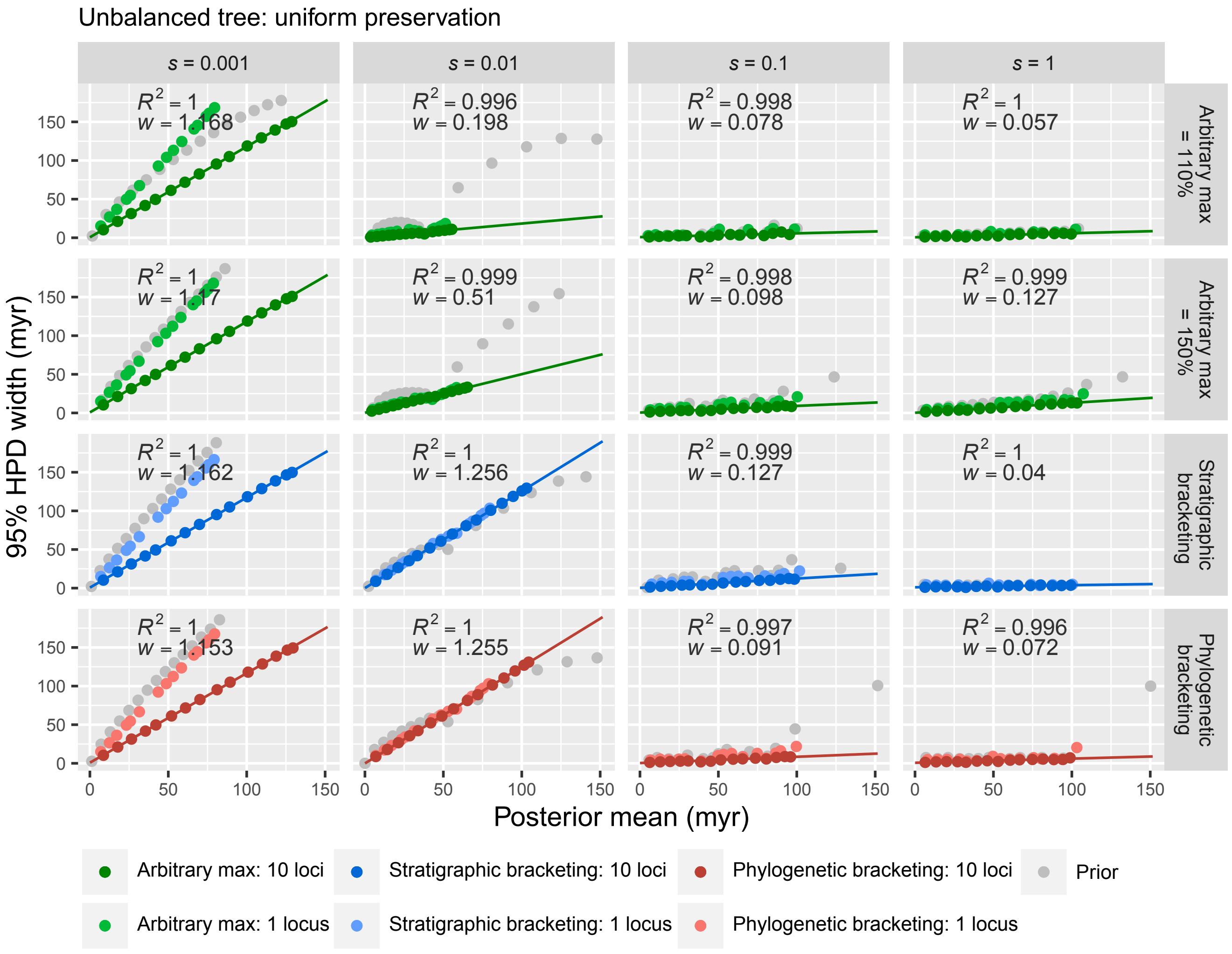

Unbalanced tree: uniform preservation
s = 0.001
s = 0.01
s = 0.1
s = 1
Arbitrary max = 110%
Arbitrary max = 150%
Stratigraphic bracketing
Phylogenetic bracketing
95% HPD width (myr)
Posterior mean (myr)
R2 = 1 w = 1.168
R2 = 0.996 w = 0.198
R2 = 0.998 w = 0.078
R2 = 1 w = 0.057
R2 = 1 w = 1.17
R2 = 0.999 w = 0.51
R2 = 0.998 w = 0.098
R2 = 0.999 w = 0.127
R2 = 1 w = 1.162
R2 = 1 w = 1.256
R2 = 0.999 w = 0.127
R2 = 1 w = 0.04
R2 = 1 w = 1.153
R2 = 1 w = 1.255
R2 = 0.997 w = 0.091
R2 = 0.996 w = 0.072
Arbitrary max: 10 loci
Stratigraphic bracketing: 10 loci
Phylogenetic bracketing: 10 loci
Prior
Arbitrary max: 1 locus
Stratigraphic bracketing: 1 locus
Phylogenetic bracketing: 1 locus

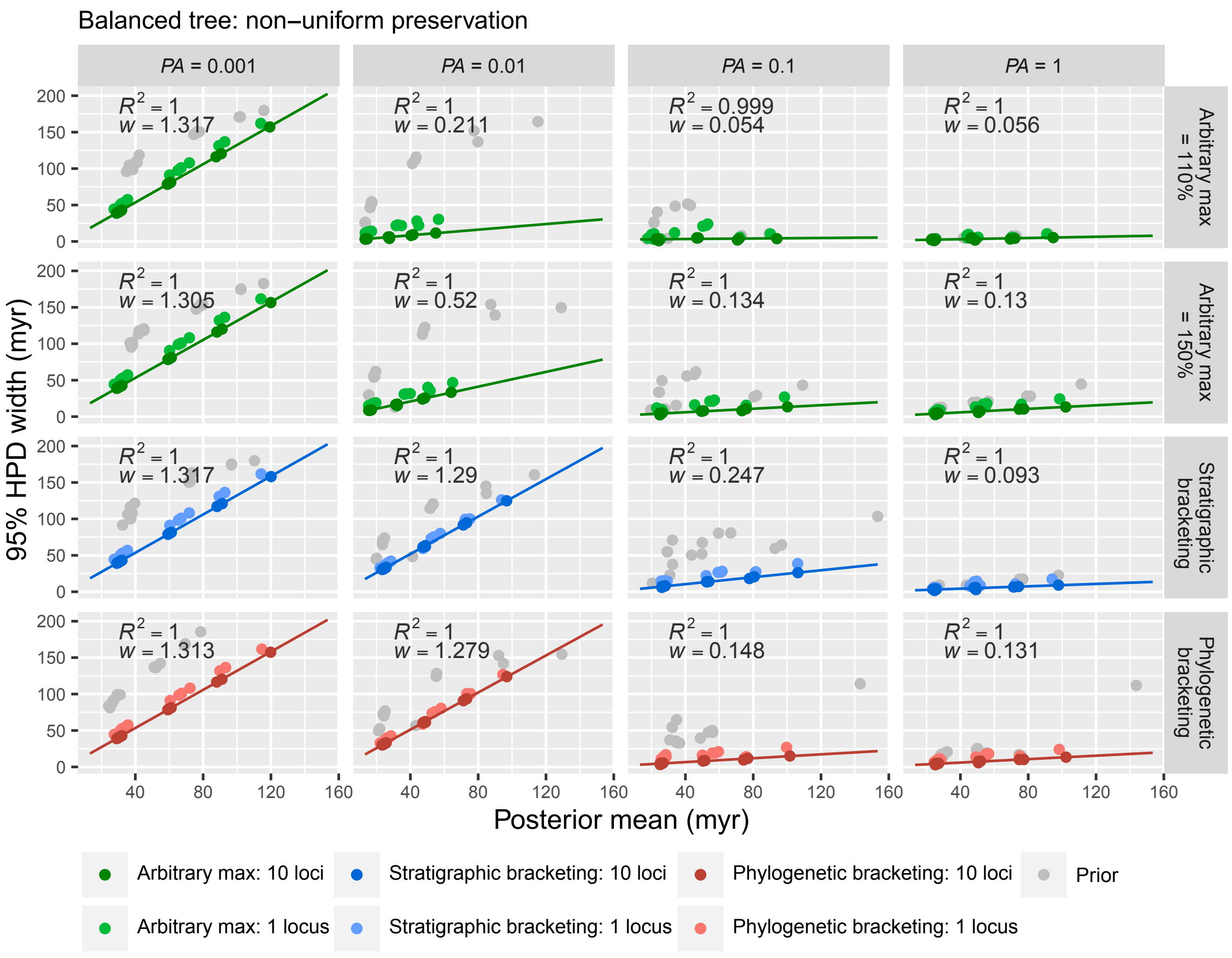
Balanced tree: non–uniform preservation
PA = 0.001
PA = 0.01
PA = 0.1
PA = 1
Arbitrary max = 110%
Arbitrary max = 150%
Stratigraphic bracketing
Phylogenetic bracketing
R2 = 1 w = 1.317
R2 = 1 w = 0.211
R2 = 0.999 w = 0.054
R2 = 1 w = 0.056
R2 = 1 w = 1.305
R2 = 1 w = 0.52
R2 = 1 w = 0.134
R2 = 1 w = 0.13
R2 = 1 w = 1.317
R2 = 1 w = 1.29
R2 = 1 w = 0.247
R2 = 1 w = 0.093
R2 = 1 w = 1.313
R2 = 1 w = 1.279
R2 = 1 w = 0.148
R2 = 1 w = 0.131
95% HPD width (myr)
Posterior mean (myr)
Arbitrary max: 10 loci
Stratigraphic bracketing: 10 loci
Phylogenetic bracketing: 10 loci
Prior
Arbitrary max: 1 locus
Stratigraphic bracketing: 1 locus
Phylogenetic bracketing: 1 locus

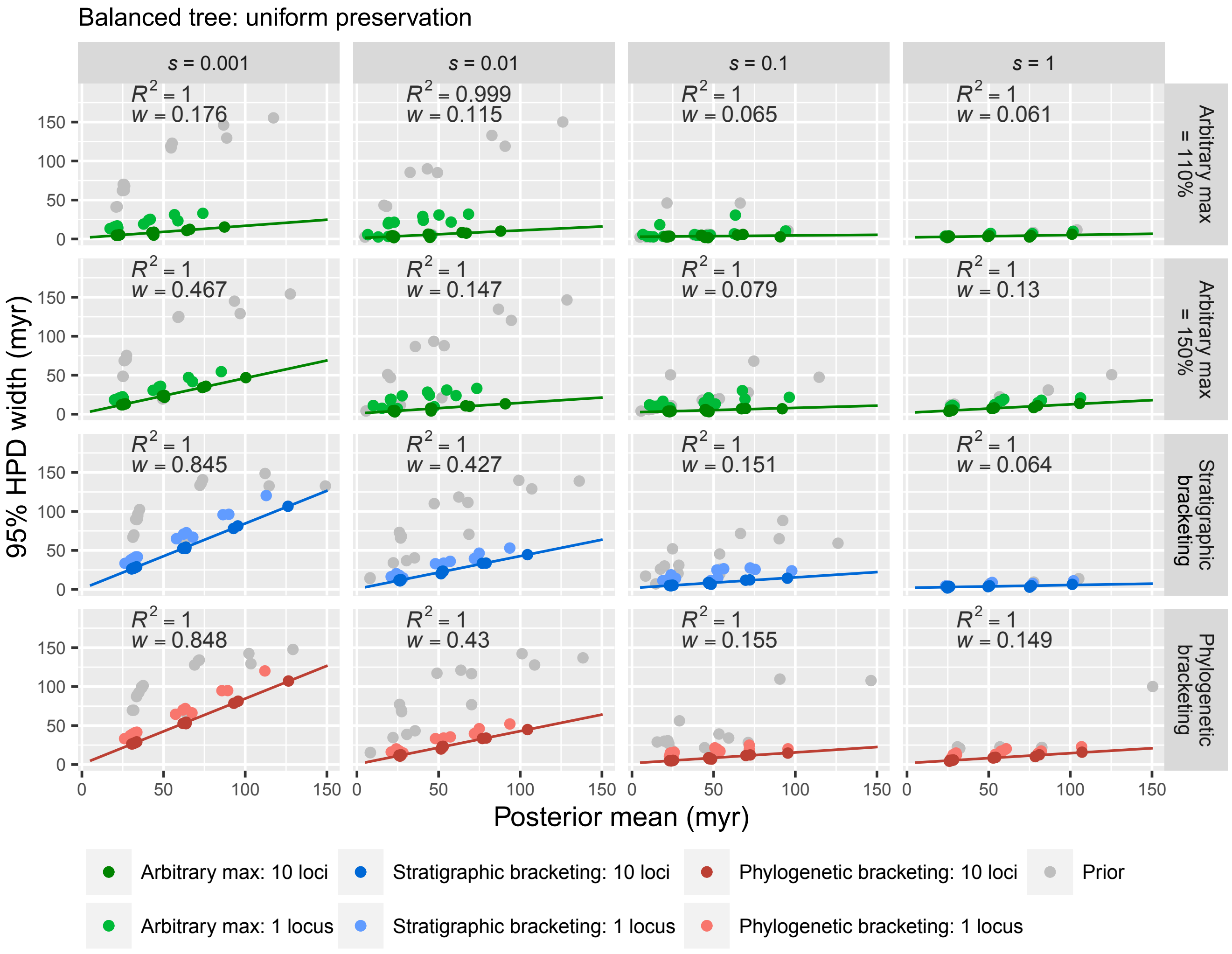

Balanced tree: uniform preservation
s = 0.001
s = 0.01
s = 0.1
s = 1
Arbitrary max = 110%
Arbitrary max = 150%
Stratigraphic bracketing
Phylogenetic bracketing
R2 = 1
w = 0.176
R2 = 0.999
w = 0.115
R2 = 1
w = 0.065
R2 = 1
w = 0.061
R2 = 1
w = 0.467
R2 = 1
w = 0.147
R2 = 1
w = 0.079
R2 = 1
w = 0.13
R2 = 1
w = 0.845
R2 = 1
w = 0.427
R2 = 1
w = 0.151
R2 = 1
w = 0.064
R2 = 1
w = 0.848
R2 = 1
w = 0.43
R2 = 1
w = 0.155
R2 = 1
w = 0.149
95% HPD width (myr)
Posterior mean (myr)
0
50
100
150
Arbitrary max: 10 loci
Arbitrary max: 1 locus
Stratigraphic bracketing: 10 loci
Stratigraphic bracketing: 1 locus
Phylogenetic bracketing: 10 loci
Phylogenetic bracketing: 1 locus
Prior

Table S1: Accuracy and precision (root mean square error, RMSE, coverage probability, CP, or interval width, $\hat{w}$) of the calibrations, and the prior and posterior time estimates obtained for the balanced tree under the uniform preservation model

				Prior and posterior estimates														
	Fossil calibrations			Prior (no data)			1 loci (1000 bases)			2 loci (2000 bases)			10 loci (10000 bases)			20 loci (20000 bases)		
Sampling (s)	Calibrated nodes (%)	CP	$\hat{w}$	RMSE	CP	$\hat{w}$	RMSE	CP	$\hat{w}$	RMSE	CP	$\hat{w}$	RMSE	CP	$\hat{w}$	RMSE	CP	$\hat{w}$
Stratigraphic bracketing – skew-t priors																		
1	1	1	0.173	0.015	1	0.15	0.021	0.987	0.138	0.024	0.98	0.128	0.018	0.983	0.092	0.015	0.977	0.076
0.1	0.933	0.954	0.742	0.157	0.874	0.825	0.08	0.971	0.413	0.062	0.97	0.303	0.036	0.975	0.178	0.031	0.984	0.154
0.01	0.263	1	0.974	0.328	0.975	2.308	0.235	0.993	0.974	0.202	0.985	0.89	0.167	0.998	0.797	0.162	1	0.778
0.001	0.088	1	0.758	0.378	0.997	3.146	0.359	0.973	1.599	0.274	0.984	1.541	0.231	1	1.47	0.213	1	1.468
Stratigraphic bracketing – uniform priors																		
1	1	1	0.173	0.045	1	0.177	0.051	0.948	0.167	0.052	0.875	0.156	0.05	0.737	0.124	0.046	0.725	0.113
0.1	0.933	0.954	0.742	0.288	0.864	1.052	0.156	0.869	0.505	0.125	0.837	0.37	0.118	0.668	0.21	0.115	0.605	0.182
0.01	0.263	1	0.974	0.454	0.975	2.637	0.343	0.959	1.19	0.31	0.969	1.109	0.278	0.982	1.037	0.27	0.994	1.028
0.001	0.088	1	0.758	0.459	0.996	3.296	0.376	0.967	1.633	0.296	0.99	1.574	0.255	0.997	1.498	0.239	1	1.496
Phylogenetic bracketing – skew-t priors																		
1	1	1	0.589	0.135	0.982	0.676	0.132	0.884	0.36	0.116	0.804	0.289	0.074	0.758	0.187	0.061	0.815	0.169
0.1	0.933	1	0.751	0.098	0.983	0.795	0.07	0.978	0.37	0.059	0.971	0.28	0.034	0.962	0.162	0.029	0.966	0.138
0.01	0.263	1	1.006	0.302	0.985	2.259	0.227	0.982	0.917	0.192	0.981	0.829	0.162	0.985	0.732	0.159	0.989	0.712
0.001	0.088	1	1.379	0.386	0.997	3.196	0.359	0.974	1.597	0.273	0.983	1.54	0.232	1	1.469	0.213	1	1.466
Phylogenetic bracketing – uniform priors																		
1	1	1	0.589	0.24	0.961	0.809	0.274	0.439	0.506	0.261	0.274	0.412	0.247	0.014	0.305	0.247	0	0.288
0.1	0.933	1	0.751	0.167	0.988	0.952	0.165	0.812	0.469	0.153	0.725	0.365	0.154	0.515	0.25	0.154	0.479	0.23
0.01	0.263	1	1.006	0.431	0.986	2.544	0.337	0.93	1.104	0.314	0.916	1.01	0.291	0.916	0.928	0.288	0.919	0.919
0.001	0.088	1	1.379	0.458	0.995	3.267	0.375	0.97	1.63	0.295	0.99	1.57	0.254	0.999	1.496	0.238	1	1.493
Arbitrary maxima (110%) – skew-t priors																		
1	1	1	0.158	0.005	1	0.136	0.014	0.965	0.128	0.019	0.963	0.119	0.016	0.975	0.088	0.015	0.971	0.073
0.1	0.933	0.421	0.189	0.204	0.401	0.199	0.195	0.347	0.161	0.175	0.296	0.161	0.085	0.265	0.099	0.069	0.276	0.077
0.01	0.263	0.444	0.215	0.248	0.823	1.491	0.278	0.47	0.442	0.265	0.337	0.299	0.226	0.265	0.173	0.221	0.246	0.153
0.001	0.088	0.811	0.225	0.384	0.978	3.013	0.373	0.86	1.369	0.304	0.827	1.292	0.277	0.781	1.195	0.271	0.777	1.215
Arbitrary maxima (125%) – skew-t priors																		
1	1	1	0.285	0.052	1	0.271	0.057	0.989	0.218	0.054	0.967	0.189	0.033	0.939	0.119	0.024	0.949	0.098
0.1	0.933	0.694	0.316	0.177	0.609	0.304	0.155	0.587	0.247	0.123	0.585	0.218	0.059	0.611	0.114	0.05	0.591	0.087
0.01	0.263	0.604	0.341	0.21	0.855	1.558	0.25	0.565	0.47	0.232	0.467	0.331	0.207	0.405	0.213	0.207	0.391	0.19
0.001	0.088	0.894	0.357	0.367	0.977	2.99	0.367	0.881	1.388	0.295	0.862	1.311	0.268	0.832	1.22	0.254	0.825	1.213

Continued on the next page

Table S1 – Continued from the previous page

				Prior and posterior estimates														
	Fossil calibrations			Prior (no data)			1 loci (1000 bases)			2 loci (2000 bases)			10 loci (10000 bases)			20 loci (20000 bases)		
Sampling (s)	Calibrated nodes (%)	CP	$\hat{w}$	RMSE	CP	$\hat{w}$	RMSE	CP	$\hat{w}$	RMSE	CP	$\hat{w}$	RMSE	CP	$\hat{w}$	RMSE	CP	$\hat{w}$
Arbitrary maxima (150%) – skew-t priors																		
1	1	1	0.457	0.131	1	0.47	0.119	0.915	0.316	0.103	0.851	0.26	0.062	0.807	0.166	0.049	0.868	0.146
0.1	0.933	0.802	0.49	0.148	0.783	0.461	0.108	0.811	0.337	0.075	0.853	0.25	0.042	0.854	0.132	0.037	0.839	0.105
0.01	0.263	0.721	0.516	0.195	0.905	1.695	0.218	0.719	0.518	0.204	0.655	0.393	0.184	0.603	0.284	0.186	0.574	0.262
0.001	0.088	0.924	0.528	0.411	0.985	3.057	0.361	0.904	1.425	0.286	0.905	1.351	0.256	0.913	1.263	0.242	0.909	1.258
Arbitrary maxima (175%) – skew-t priors																		
1	1	1	0.605	0.202	0.933	0.669	0.174	0.811	0.401	0.149	0.721	0.327	0.098	0.685	0.229	0.084	0.757	0.215
0.1	0.933	0.895	0.635	0.141	0.843	0.613	0.087	0.92	0.391	0.063	0.937	0.279	0.036	0.951	0.156	0.031	0.951	0.13
0.01	0.263	0.83	0.659	0.212	0.927	1.805	0.205	0.817	0.582	0.19	0.783	0.464	0.169	0.767	0.362	0.172	0.741	0.342
0.001	0.088	0.947	0.669	0.398	0.994	3.071	0.361	0.911	1.464	0.282	0.926	1.391	0.251	0.93	1.307	0.235	0.933	1.301

s is the probability of sampling during each interval under the uniform preservation model. % is the proportion of calibrated nodes across all simulated replicates. CP is the coverage probability across all replicates. RMSE is the relative root mean squared error and $\hat{w}$ is the relative confidence interval width, both averaged across all replicates. The tree is shown in Fig. 1.

Table S2: Accuracy and precision (root mean square error, RMSE, coverage probability, CP, or interval width, $\hat{w}$) of the calibrations, and the prior and posterior time estimates obtained for the balanced tree under the non-uniform preservation model

				Prior and posterior estimates														
	Fossil calibrations			Prior (no data)			1 loci (1000 bases)			2 loci (2000 bases)			10 loci (10000 bases)			20 loci (20000 bases)		
Sampling (PA)	Calibrated nodes (%)	CP	$\hat{w}$	RMSE	CP	$\hat{w}$	RMSE	CP	$\hat{w}$	RMSE	CP	$\hat{w}$	RMSE	CP	$\hat{w}$	RMSE	CP	$\hat{w}$
Stratigraphic bracketing – skew-t priors																		
1	1	0.923	0.274	0.149	0.811	0.237	0.087	0.939	0.199	0.063	0.945	0.172	0.03	0.954	0.109	0.025	0.937	0.086
0.1	0.723	1	0.909	0.176	0.975	1.308	0.116	0.989	0.504	0.09	0.989	0.395	0.061	0.991	0.294	0.055	0.995	0.274
0.01	0.161	1	1.009	0.369	1	2.768	0.287	0.986	1.259	0.231	0.995	1.164	0.183	0.997	1.1	0.173	0.999	1.088
0.001	0.077	1	0.817	0.424	0.999	3.294	0.365	0.98	1.679	0.292	0.989	1.613	0.225	1	1.541	0.21	1	1.535
Stratigraphic bracketing – uniform priors																		
1	1	0.923	0.274	0.11	0.889	0.277	0.06	0.912	0.221	0.047	0.907	0.184	0.033	0.885	0.104	0.03	0.866	0.08
0.1	0.723	1	0.909	0.401	0.941	1.732	0.288	0.869	0.729	0.259	0.837	0.616	0.239	0.848	0.528	0.232	0.873	0.511
0.01	0.161	1	1.009	0.482	0.999	3.049	0.351	0.98	1.409	0.306	0.989	1.316	0.267	0.996	1.258	0.257	0.999	1.252
0.001	0.077	1	0.817	0.435	1	3.314	0.375	0.976	1.689	0.303	0.989	1.625	0.239	1	1.551	0.222	1	1.545
Phylogenetic bracketing – skew-t priors																		
1	1	1	0.552	0.039	0.999	0.569	0.084	0.982	0.32	0.072	0.959	0.255	0.042	0.937	0.155	0.031	0.953	0.131
0.1	0.723	1	0.745	0.103	0.991	1.046	0.077	0.957	0.377	0.067	0.933	0.286	0.05	0.878	0.183	0.047	0.887	0.16
0.01	0.161	1	0.83	0.364	0.999	2.742	0.28	0.977	1.233	0.225	0.989	1.135	0.182	0.986	1.07	0.171	0.989	1.057
0.001	0.077	1	1.061	0.408	0.999	3.257	0.366	0.979	1.678	0.294	0.989	1.613	0.226	1	1.541	0.211	1	1.534
Phylogenetic bracketing – uniform priors																		
1	1	1	0.552	0.149	0.998	0.674	0.191	0.65	0.429	0.173	0.551	0.348	0.15	0.283	0.235	0.148	0.18	0.22
0.1	0.723	1	0.745	0.193	0.991	1.258	0.144	0.932	0.465	0.131	0.877	0.368	0.121	0.849	0.268	0.122	0.835	0.248
0.01	0.161	1	0.83	0.456	0.999	2.993	0.347	0.974	1.369	0.302	0.987	1.266	0.268	0.992	1.2	0.258	0.999	1.192
0.001	0.077	1	1.061	0.425	0.999	3.267	0.376	0.979	1.692	0.306	0.989	1.624	0.241	0.995	1.551	0.225	0.997	1.546
Arbitrary maxima (110%) – skew-t priors																		
1	1	0.692	0.158	0.183	0.679	0.127	0.154	0.559	0.12	0.117	0.588	0.116	0.052	0.641	0.085	0.04	0.642	0.069
0.1	0.723	0.256	0.175	0.298	0.449	0.426	0.255	0.223	0.204	0.222	0.137	0.166	0.13	0.066	0.094	0.11	0.061	0.073
0.01	0.161	0.481	0.176	0.3	0.909	2.169	0.302	0.596	0.74	0.269	0.453	0.612	0.24	0.311	0.508	0.232	0.321	0.5
0.001	0.077	0.896	0.207	0.401	0.989	3.163	0.358	0.921	1.555	0.301	0.907	1.477	0.25	0.903	1.387	0.239	0.908	1.389
Arbitrary maxima (125%) – skew-t priors																		
1	1	0.957	0.289	0.145	0.871	0.255	0.083	0.955	0.209	0.06	0.953	0.179	0.029	0.957	0.112	0.024	0.948	0.089
0.1	0.723	0.593	0.304	0.263	0.622	0.523	0.194	0.427	0.258	0.16	0.322	0.198	0.102	0.246	0.109	0.089	0.237	0.087
0.01	0.161	0.693	0.305	0.263	0.913	2.143	0.28	0.698	0.781	0.244	0.613	0.654	0.219	0.5	0.556	0.21	0.492	0.541
0.001	0.077	0.93	0.324	0.392	0.991	3.172	0.356	0.941	1.566	0.299	0.923	1.487	0.244	0.917	1.401	0.235	0.917	1.392

Continued on the next page

Table S2 – Continued from the previous page

				Prior and posterior estimates														
	Fossil calibrations			Prior (no data)			1 loci (1000 bases)			2 loci (2000 bases)			10 loci (10000 bases)			20 loci (20000 bases)		
Sampling (PA)	Calibrated nodes (%)	CP	$\hat{w}$	RMSE	CP	$\hat{w}$	RMSE	CP	$\hat{w}$	RMSE	CP	$\hat{w}$	RMSE	CP	$\hat{w}$	RMSE	CP	$\hat{w}$
Arbitrary maxima (150%) – skew-t priors																		
1	1	1	0.46	0.089	1	0.439	0.069	0.987	0.298	0.061	0.972	0.242	0.035	0.962	0.146	0.026	0.967	0.121
0.1	0.723	0.815	0.477	0.208	0.813	0.673	0.128	0.762	0.314	0.108	0.68	0.232	0.076	0.553	0.133	0.069	0.55	0.109
0.01	0.161	0.851	0.477	0.288	0.961	2.263	0.258	0.843	0.855	0.215	0.789	0.735	0.188	0.739	0.645	0.179	0.733	0.631
0.001	0.077	0.948	0.506	0.372	0.981	3.117	0.354	0.955	1.589	0.294	0.948	1.511	0.235	0.94	1.426	0.226	0.94	1.417
Arbitrary maxima (175%) – skew-t priors																		
1	1	1	0.606	0.06	0.997	0.612	0.111	0.946	0.372	0.096	0.917	0.299	0.06	0.897	0.191	0.047	0.933	0.17
0.1	0.723	0.946	0.624	0.154	0.921	0.821	0.091	0.933	0.359	0.078	0.909	0.27	0.058	0.835	0.166	0.053	0.831	0.143
0.01	0.161	0.959	0.624	0.317	0.979	2.393	0.248	0.921	0.941	0.197	0.915	0.826	0.165	0.894	0.742	0.155	0.894	0.731
0.001	0.077	0.965	0.65	0.413	0.996	3.202	0.356	0.96	1.61	0.293	0.969	1.535	0.232	0.96	1.451	0.22	0.959	1.443

PA is the peak abundance parameter that determines the probability of sampling during each interval under the non-uniform preservation model. % is the proportion of calibrated nodes across all simulated replicates. CP is the coverage probability across all replicates. RMSE is the relative root mean squared error and $\hat{w}$ is the relative confidence interval width, both averaged across all replicates. The tree is shown in Fig. 1.

Table S3: Accuracy and precision (root mean square error, RMSE, coverage probability, CP, or interval width, $\hat{w}$) of the calibrations, and the prior and posterior time estimates obtained for the unbalanced tree under the uniform preservation model

				Prior and posterior estimates														
	Fossil calibrations			Prior (no data)			1 loci (1000 bases)			2 loci (2000 bases)			10 loci (10000 bases)			20 loci (20000 bases)		
Sampling (s)	Calibrated nodes (%)	CP	$\hat{w}$	RMSE	CP	$\hat{w}$	RMSE	CP	$\hat{w}$	RMSE	CP	$\hat{w}$	RMSE	CP	$\hat{w}$	RMSE	CP	$\hat{w}$
Stratigraphic bracketing – skew-t priors																		
1	1	1	0.163	0.011	1	0.141	0.012	0.981	0.11	0.011	0.96	0.094	0.01	0.893	0.062	0.01	0.84	0.051
0.1	0.887	0.986	0.765	0.056	0.887	0.467	0.054	0.97	0.323	0.051	0.96	0.255	0.038	0.979	0.178	0.034	0.981	0.162
0.01	0.304	0.998	1.131	0.306	0.767	0.986	0.183	0.964	0.825	0.185	0.967	0.768	0.189	0.955	0.709	0.189	0.965	0.697
0.001	0.09	1	0.914	0.268	0.896	1.841	0.319	0.954	1.454	0.27	0.98	1.449	0.232	0.999	1.422	0.22	1	1.428
Stratigraphic bracketing – uniform priors																		
1	1	1	0.163	0.016	1	0.173	0.016	0.986	0.137	0.015	0.977	0.119	0.013	0.939	0.077	0.012	0.92	0.062
0.1	0.887	0.986	0.765	0.15	0.792	0.572	0.14	0.772	0.392	0.143	0.733	0.321	0.139	0.691	0.243	0.14	0.71	0.229
0.01	0.304	0.998	1.131	0.347	0.793	1.14	0.267	0.963	1.032	0.272	0.959	0.994	0.278	0.967	0.956	0.279	0.976	0.953
0.001	0.09	1	0.914	0.267	0.92	1.874	0.321	0.965	1.485	0.272	0.987	1.478	0.239	0.999	1.45	0.23	0.999	1.452
Phylogenetic bracketing – skew-t priors																		
1	1	1	0.285	0.014	0.983	0.279	0.016	0.995	0.173	0.015	0.989	0.135	0.01	0.959	0.08	0.009	0.939	0.063
0.1	0.887	0.859	0.506	0.078	0.784	0.422	0.067	0.786	0.227	0.057	0.77	0.168	0.042	0.642	0.091	0.037	0.54	0.071
0.01	0.304	0.796	0.889	0.316	0.707	0.871	0.231	0.754	0.558	0.209	0.737	0.482	0.206	0.699	0.404	0.205	0.685	0.386
0.001	0.09	0.978	1.276	0.293	0.879	1.771	0.32	0.947	1.432	0.272	0.959	1.424	0.235	0.979	1.396	0.223	0.979	1.402
Phylogenetic bracketing – uniform priors																		
1	1	1	0.285	0.035	0.966	0.338	0.038	0.863	0.221	0.04	0.823	0.175	0.036	0.772	0.106	0.033	0.78	0.088
0.1	0.887	0.859	0.506	0.077	0.794	0.464	0.072	0.793	0.239	0.069	0.755	0.172	0.064	0.545	0.089	0.059	0.453	0.07
0.01	0.304	0.796	0.889	0.329	0.712	0.939	0.307	0.661	0.564	0.313	0.609	0.486	0.309	0.552	0.408	0.307	0.539	0.393
0.001	0.09	0.978	1.276	0.261	0.915	1.875	0.324	0.939	1.448	0.277	0.957	1.434	0.243	0.97	1.403	0.234	0.969	1.405
Arbitrary maxima (110%) – skew-t priors																		
1	1	0.993	0.166	0.017	0.96	0.134	0.018	0.975	0.12	0.016	0.962	0.107	0.011	0.922	0.073	0.009	0.902	0.06
0.1	0.887	0.483	0.18	0.127	0.365	0.188	0.092	0.351	0.15	0.09	0.331	0.128	0.057	0.245	0.078	0.077	0.208	0.058
0.01	0.304	0.342	0.205	0.502	0.319	0.437	0.424	0.281	0.264	0.389	0.26	0.218	0.351	0.187	0.135	0.358	0.141	0.088
0.001	0.09	0.763	0.214	0.315	0.795	1.635	0.374	0.765	1.175	0.34	0.753	1.143	0.332	0.737	1.076	0.302	0.772	1.117
Arbitrary maxima (125%) – skew-t priors																		
1	1	1	0.293	0.048	0.947	0.226	0.045	0.953	0.184	0.043	0.929	0.158	0.028	0.897	0.1	0.023	0.904	0.084
0.1	0.887	0.684	0.306	0.085	0.603	0.245	0.068	0.643	0.195	0.057	0.665	0.157	0.042	0.559	0.088	0.038	0.482	0.07
0.01	0.304	0.461	0.326	0.463	0.4	0.508	0.374	0.4	0.31	0.34	0.373	0.237	0.318	0.339	0.156	0.322	0.319	0.14
0.001	0.09	0.815	0.338	0.313	0.811	1.654	0.365	0.795	1.193	0.33	0.794	1.167	0.321	0.792	1.102	0.315	0.789	1.099

Continued on the next page

Table S3 – Continued from the previous page

	Fossil calibrations			Prior and posterior estimates														
				Prior (no data)			1 loci (1000 bases)			2 loci (2000 bases)			10 loci (10000 bases)			20 loci (20000 bases)		
Sampling (s)	Calibrated nodes (%)	CP	$\hat{w}$	RMSE	CP	$\hat{w}$	RMSE	CP	$\hat{w}$	RMSE	CP	$\hat{w}$	RMSE	CP	$\hat{w}$	RMSE	CP	$\hat{w}$
Arbitrary maxima (150%) – skew-t priors																		
1	1	1	0.466	0.091	0.911	0.329	0.088	0.85	0.263	0.083	0.811	0.218	0.056	0.827	0.149	0.05	0.835	0.136
0.1	0.887	0.804	0.48	0.064	0.776	0.337	0.047	0.868	0.245	0.042	0.881	0.191	0.031	0.834	0.11	0.027	0.797	0.091
0.01	0.304	0.583	0.502	0.41	0.489	0.55	0.321	0.515	0.353	0.3	0.494	0.284	0.282	0.465	0.205	0.291	0.451	0.186
0.001	0.09	0.837	0.513	0.299	0.827	1.679	0.354	0.805	1.231	0.318	0.814	1.205	0.308	0.813	1.143	0.3	0.813	1.14
Arbitrary maxima (175%) – skew-t priors																		
1	1	1	0.61	0.14	0.881	0.434	0.133	0.724	0.332	0.123	0.671	0.276	0.091	0.739	0.215	0.086	0.769	0.206
0.1	0.887	0.873	0.624	0.063	0.819	0.413	0.046	0.939	0.285	0.044	0.937	0.22	0.029	0.933	0.137	0.025	0.927	0.119
0.01	0.304	0.649	0.645	0.378	0.539	0.623	0.303	0.599	0.4	0.278	0.595	0.33	0.26	0.59	0.251	0.26	0.581	0.235
0.001	0.09	0.837	0.654	0.278	0.835	1.662	0.346	0.81	1.268	0.308	0.825	1.243	0.296	0.819	1.182	0.288	0.819	1.18

s is the probability of sampling during each interval under the uniform preservation model. % is the proportion of calibrated nodes across all simulated replicates. CP is the coverage probability across all replicates. RMSE is the relative root mean squared error and $\hat{w}$ is the relative confidence interval width, both averaged across all replicates. The tree is shown in Fig. 1.

Table S4: Accuracy and precision (root mean square error, RMSE, coverage probability, CP, or interval width, $\hat{w}$) of the calibrations, and the prior and posterior time estimates obtained for the unbalanced tree under the non-uniform preservation model

				Prior and posterior estimates														
	Fossil calibrations			Prior (no data)			1 loci (1000 bases)			2 loci (2000 bases)			10 loci (10000 bases)			20 loci (20000 bases)		
Sampling (PA)	Calibrated nodes (%)	CP	$\hat{w}$	RMSE	CP	$\hat{w}$	RMSE	CP	$\hat{w}$	RMSE	CP	$\hat{w}$	RMSE	CP	$\hat{w}$	RMSE	CP	$\hat{w}$
Stratigraphic bracketing – skew-t priors																		
1	1	0.923	0.274	0.149	0.811	0.237	0.087	0.939	0.199	0.063	0.945	0.172	0.03	0.954	0.109	0.025	0.937	0.086
0.1	0.723	1	0.909	0.176	0.975	1.308	0.116	0.989	0.504	0.09	0.989	0.395	0.061	0.991	0.294	0.055	0.995	0.274
0.01	0.161	1	1.009	0.369	1	2.768	0.287	0.986	1.259	0.231	0.995	1.164	0.183	0.997	1.1	0.173	0.999	1.088
0.001	0.077	1	0.817	0.424	0.999	3.294	0.365	0.98	1.679	0.292	0.989	1.613	0.225	1	1.541	0.21	1	1.535
Stratigraphic bracketing – uniform priors																		
1	1	0.923	0.274	0.11	0.889	0.277	0.06	0.912	0.221	0.047	0.907	0.184	0.033	0.885	0.104	0.03	0.866	0.08
0.1	0.723	1	0.909	0.401	0.941	1.732	0.288	0.869	0.729	0.259	0.837	0.616	0.239	0.848	0.528	0.232	0.873	0.511
0.01	0.161	1	1.009	0.482	0.999	3.049	0.351	0.98	1.409	0.306	0.989	1.316	0.267	0.996	1.258	0.257	0.999	1.252
0.001	0.077	1	0.817	0.435	1	3.314	0.375	0.976	1.689	0.303	0.989	1.625	0.239	1	1.551	0.222	1	1.545
Phylogenetic bracketing – skew-t priors																		
1	1	1	0.552	0.039	0.999	0.569	0.084	0.982	0.32	0.072	0.959	0.255	0.042	0.937	0.155	0.031	0.953	0.131
0.1	0.723	1	0.745	0.103	0.991	1.046	0.077	0.957	0.377	0.067	0.933	0.286	0.05	0.878	0.183	0.047	0.887	0.16
0.01	0.161	1	0.83	0.364	0.999	2.742	0.28	0.977	1.233	0.225	0.989	1.135	0.182	0.986	1.07	0.171	0.989	1.057
0.001	0.077	1	1.061	0.408	0.999	3.257	0.366	0.979	1.678	0.294	0.989	1.613	0.226	1	1.541	0.211	1	1.534
Phylogenetic bracketing – uniform priors																		
1	1	1	0.552	0.149	0.998	0.674	0.191	0.65	0.429	0.173	0.551	0.348	0.15	0.283	0.235	0.148	0.18	0.22
0.1	0.723	1	0.745	0.193	0.991	1.258	0.144	0.932	0.465	0.131	0.877	0.368	0.121	0.849	0.268	0.122	0.835	0.248
0.01	0.161	1	0.83	0.456	0.999	2.993	0.347	0.974	1.369	0.302	0.987	1.266	0.268	0.992	1.2	0.258	0.999	1.192
0.001	0.077	1	1.061	0.425	0.999	3.267	0.376	0.979	1.692	0.306	0.989	1.624	0.241	0.995	1.551	0.225	0.997	1.546
Arbitrary maxima (110%) – skew-t priors																		
1	1	0.692	0.158	0.183	0.679	0.127	0.154	0.559	0.12	0.117	0.588	0.116	0.052	0.641	0.085	0.04	0.642	0.069
0.1	0.723	0.256	0.175	0.298	0.449	0.426	0.255	0.223	0.204	0.222	0.137	0.166	0.13	0.066	0.094	0.11	0.061	0.073
0.01	0.161	0.481	0.176	0.3	0.909	2.169	0.302	0.596	0.74	0.269	0.453	0.612	0.24	0.311	0.508	0.232	0.321	0.5
0.001	0.077	0.896	0.207	0.401	0.989	3.163	0.358	0.921	1.555	0.301	0.907	1.477	0.25	0.903	1.387	0.239	0.908	1.389
Arbitrary maxima (125%) – skew-t priors																		
1	1	0.957	0.289	0.145	0.871	0.255	0.083	0.955	0.209	0.06	0.953	0.179	0.029	0.957	0.112	0.024	0.948	0.089
0.1	0.723	0.593	0.304	0.263	0.622	0.523	0.194	0.427	0.258	0.16	0.322	0.198	0.102	0.246	0.109	0.089	0.237	0.087
0.01	0.161	0.693	0.305	0.263	0.913	2.143	0.28	0.698	0.781	0.244	0.613	0.654	0.219	0.5	0.556	0.21	0.492	0.541
0.001	0.077	0.93	0.324	0.392	0.991	3.172	0.356	0.941	1.566	0.299	0.923	1.487	0.244	0.917	1.401	0.235	0.917	1.392

Continued on the next page

Table S4 – Continued from the previous page

				Prior and posterior estimates														
	Fossil calibrations			Prior (no data)			1 loci (1000 bases)			2 loci (2000 bases)			10 loci (10000 bases)			20 loci (20000 bases)		
Sampling (PA)	Calibrated nodes (%)	CP	$\hat{w}$	RMSE	CP	$\hat{w}$	RMSE	CP	$\hat{w}$	RMSE	CP	$\hat{w}$	RMSE	CP	$\hat{w}$	RMSE	CP	$\hat{w}$
Arbitrary maxima (150%) – skew-t priors																		
1	1	1	0.46	0.089	1	0.439	0.069	0.987	0.298	0.061	0.972	0.242	0.035	0.962	0.146	0.026	0.967	0.121
0.1	0.723	0.815	0.477	0.208	0.813	0.673	0.128	0.762	0.314	0.108	0.68	0.232	0.076	0.553	0.133	0.069	0.55	0.109
0.01	0.161	0.851	0.477	0.288	0.961	2.263	0.258	0.843	0.855	0.215	0.789	0.735	0.188	0.739	0.645	0.179	0.733	0.631
0.001	0.077	0.948	0.506	0.372	0.981	3.117	0.354	0.955	1.589	0.294	0.948	1.511	0.235	0.94	1.426	0.226	0.94	1.417
Arbitrary maxima (175%) – skew-t priors																		
1	1	1	0.606	0.06	0.997	0.612	0.111	0.946	0.372	0.096	0.917	0.299	0.06	0.897	0.191	0.047	0.933	0.17
0.1	0.723	0.946	0.624	0.154	0.921	0.821	0.091	0.933	0.359	0.078	0.909	0.27	0.058	0.835	0.166	0.053	0.831	0.143
0.01	0.161	0.959	0.624	0.317	0.979	2.393	0.248	0.921	0.941	0.197	0.915	0.826	0.165	0.894	0.742	0.155	0.894	0.731
0.001	0.077	0.965	0.65	0.413	0.996	3.202	0.356	0.96	1.61	0.293	0.969	1.535	0.232	0.96	1.451	0.22	0.959	1.443

PA is the peak abundance parameter that determines the probability of sampling during each interval under the non-uniform preservation model. % is the proportion of calibrated nodes across all simulated replicates. CP is the coverage probability across all replicates. RMSE is the relative root mean squared error and $\hat{w}$ is the relative confidence interval width, both averaged across all replicates. The tree is shown in Fig. 1.
